# Genomic aberrations after short-term exposure to colibactin-producing *E. coli* transform primary colon epithelial cells

**DOI:** 10.1038/s41467-021-21162-y

**Published:** 2021-02-12

**Authors:** Amina Iftekhar, Hilmar Berger, Nassim Bouznad, Julian Heuberger, Francesco Boccellato, Ulrich Dobrindt, Heiko Hermeking, Michael Sigal, Thomas F. Meyer

**Affiliations:** 1 Department of Molecular Biology, Max Planck Institute for Infection Biology, Berlin, Germany; 2 Laboratory of Infection Oncology, Institute of Clinical Molecular Biology, Christian Albrechts University of Kiel and University Hospital Schleswig Holstein – Campus Kiel, Kiel, Germany; 3 Department of Internal Medicine, Gastroenterology and Hepatology, Charité University Medicine, Berlin, Germany; 4 Experimental and Molecular Pathology, Institute of Pathology, Ludwig Maximilians University, München, Germany; 5 Ludwig Institute for Cancer Research, University of Oxford, Oxford, UK; 6 Institute of Hygiene, University of Münster, Münster, Germany; 7 German Cancer Consortium (DKTK), Partner Site München, München, Germany; 8 German Cancer Research Center (DKFZ), Heidelberg, Germany; 9 Berlin Institute for Medical Systems Biology, Max Delbrück Center for Molecular Medicine, Berlin, Germany

**Keywords:** Cancer genomics, Bacteriology, Colorectal cancer

## Abstract

Genotoxic colibactin-producing *pks*+ *Escherichia coli* induce DNA double-strand breaks, mutations, and promote tumor development in mouse models of colorectal cancer (CRC). Colibactin’s distinct mutational signature is reflected in human CRC, suggesting a causal link. Here, we investigate its transformation potential using organoids from primary murine colon epithelial cells. Organoids recovered from short-term infection with *pks*+ *E. coli* show characteristics of CRC cells, e.g., enhanced proliferation, Wnt-independence, and impaired differentiation. Sequence analysis of Wnt-independent organoids reveals an enhanced mutational burden, including chromosomal aberrations typical of genomic instability. Although we do not find classic Wnt-signaling mutations, we identify several mutations in genes related to p53-signaling, including *miR-34a*. Knockout of *Trp53* or *miR-34* in organoids results in Wnt-independence, corroborating a functional interplay between the p53 and Wnt pathways. We propose larger chromosomal alterations and aneuploidy as the basis of transformation in these organoids, consistent with the early appearance of chromosomal instability in CRC.

## Abstract (web summary)

Colibactin-producing *pks+* *Escherichia coli* are frequent constituents of the human intestinal microbiota. Here, the authors show that short exposure of cells to *pks+* *E. coli* induces chromosomal aberrations, genomic instability, and multiple features of transformation reminiscent of colorectal cancer.

## Introduction

Colorectal cancer (CRC) is the second leading cause of cancer-related mortality worldwide. The genetic landscape of mutations that drive carcinogenesis is well explored and involves the Wnt, KRAS, TGF-β, and p53 pathways^1^. Combinations of mutations in these pathways are responsible for the transformation of epithelial cells^2,3^, and constitutively active Wnt signaling is considered to be a crucial feature of almost all CRCs^4^. Wnt signaling drives proliferation of colon cells and is physiologically active only in the stem cell compartment, while differentiated cells lack Wnt signaling^5^. Experimental and patient-derived data indicate that constitutively active Wnt signaling is an early event in the CRC cascade, often achieved by mutation of *APC* and subsequent loss of heterozygosity (LOH)^1,6^. Although, this holds true for sporadic cases of CRC, cases driven by chronic inflammatory disorders, such as ulcerative colitis or Crohn’s colitis, are associated with a distinct mutational profile, in which mutations in the p53 pathway are thought to occur as an early event^7–10^. While many of the mutations in driver genes are due to single nucleotide substitutions and small insertion and deletions, chromosomal aberrations are frequent in CRC and found in the majority of cases^4^. Chromosomal instability (CIN) is often attributed to cell-intrinsic causes like spindle assembly checkpoint defects^11^, but the exact mechanisms leading to CIN are mostly unknown.

Recent data suggest that environmental factors are important drivers of CRC and that the colonic microbiota plays an important role in both the development and progression of CRC^12–14^. In addition to microbial factors that induce inflammation and therefore contribute to carcinogenesis by triggering oxidative stress or changes in the stem cell niche, certain bacteria that colonize the gut appear to directly affect the genomic integrity of epithelial cells through genotoxins. In this context, colibactin is a prominent genotoxin produced by several Enterobacteriaceae members, such as *E. coli* belonging to phylogenetic group B2. This toxin is a polyketide/nonribosomal peptide hybrid, encoded by the *pks* pathogenicity island. Infection of eukaryotic cell lines with *pks*+ *E. coli* was shown to cause DNA double-strand breaks (DSBs), leading to megalocytosis and cell cycle arrest^15^. Short exposure of an intestinal epithelial cell line to *pks*+ *E. coli* has been reported to cause genomic instability and anchorage-independent growth^16^. Significant enrichment of *pks* gene clusters has been observed in CRC metagenomes^13^. An increase in colonic mucosa-associated *E. coli* that possess the *pks* gene has been observed in inflammatory bowel disease (IBD), familial adenomatous polyposis (FAP), and CRC patients^17–19^. Colibactin alkylates DNA through covalent modifications and forms stable adenine-colibactin adducts in cultured mammalian cells and in vivo^20–22^. It has also been shown to leave a specific mutational signature in CRC patients^23,24^. Moreover, *pks*+ *E. coli* enhance tumorigenesis in different murine models of FAP and CRC, such as mice heterozygous for *Apc*^18^, interleukin-10–deficient (*Il10*^*−/−*^) germ-free mice^17^, and the azoxymethane/dextran sulfate sodium (AOM/DSS) model^25^. Notably, in these mouse models, tumors can also arise in the absence of colibactin, leaving it uncertain whether colibactin merely enhances tumorigenesis or whether it can also play a causative role. To study genomic instability caused by *pks*+ *E. coli* in vitro, infections have only been performed with transformed or immortalized cell lines, and it remains unclear whether healthy primary colon epithelial cells respond to infection in the same way.

To address this, we developed primary cell culture models derived from normal murine colon epithelial cells. We infected organoids as well as polarized monolayers derived from primary colon epithelial cells with *pks*+ *E. coli* or an isogenic mutant that is defective for colibactin synthesis. In these models, we were able to reproduce early responses to infection, including DSBs and development of megalocytosis—effects unique to strains with a functional *pks* island. Furthermore, we examined whether colibactin-induced DNA damage after short-term infection causes genomic instability, mutations, or changes in niche dependence of colon organoids. We specifically investigated Wnt-independence due to its clinical relevance. Our findings reveal that the genotoxic effect of colibactin is sufficient to rapidly cause genomic aberrations in primary colon cells and to induce multiple features of transformation reminiscent of CRC. These transformed cells did not exhibit the typical colibactin mutational signature, suggesting that improper repair of colibactin-induced cross-links after short exposure to the bacteria can lead to copy number variations (CNV) and other mutations. Accordingly, chromosomal aberrations causing aneuploidy induced by colibactin are sufficient to cause primary cell transformation and precede detectable genomic imprinting of the colibactin mutational signature. The transforming capacity of colibactin-producing *E. coli* may thus be higher than what is estimated from the presence of the mutational signature alone.

## Results

### *pks*+ *E. coli* cause DNA damage, megalocytosis, and formation of multinucleated cells in primary colon epithelial cell cultures

**Fig. 1:**
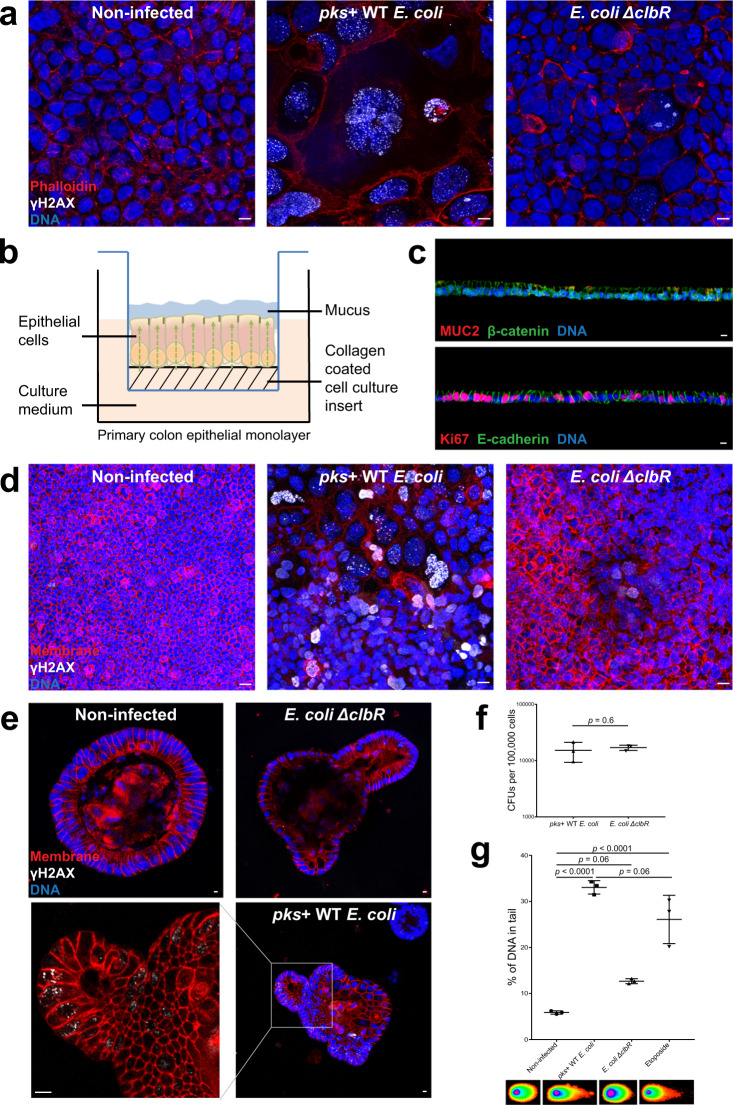
*pks*+ *E. coli* cause DNA damage, megalocytosis, and formation of multinucleated cells in primary colon epithelial cell cultures. **a** Immunofluorescence staining of Caco-2 cells showing γH2AX (white). Phalloidin (red) stains for actin filaments and Hoechst (blue) stains for DNA. Caco-2 cells infected with *pks*+ WT *E. coli* are γH2AX-positive, display megalocytosis, and are multinucleated. Less γH2AX is detected in cells infected with the mutant strain (*E. coli ΔclbR*) defective for colibactin synthesis. Image representative of three independent replicates. Scale bars: 10 μm. **b** Epithelial cells from colon organoids are sheared and seeded on collagen-coated polycarbonate filter inserts. The cells attach and form a columnar polarized monolayer. Secreted mucus accumulates on the apical side. **c** Top: Cross-section of the polarized monolayer showing expression of colonic mucus MUC2 (red) and β-catenin (green). Hoechst (blue) stains for DNA. Bottom: Cross-section of the polarized monolayer showing expression of basolateral E-cadherin (green) and the proliferation marker Ki67 (red). Hoechst (blue) stains for DNA. Image representative of three independent replicates. Scale bars: 10 μm. **d** tdTomato (red) expressing colonic monolayer immunostained for γH2AX (white); Hoechst (blue) stains for DNA. Megalocytosis, multinucleated cells, and γH2AX-positive cells are observed in monolayer infected with *pks*+ WT *E. coli*. Image representative of three independent replicates. Scale bars: 10 μm. **e** Murine colon organoids expressing membrane tdTomato (red) immunostained for γH2AX (white); Hoechst (blue) was used to stain for DNA. Cells in colon organoids are γH2AX-positive only when infected with the *pks*+ WT *E. coli*. Image representative of three independent replicates. Scale bars: 10 μm. **f** CFUs per 100,000 cells of murine colon organoids from three independent replicates. Data represent mean ± SD, *p* < 0.05, as calculated by two-sided unpaired Student’s *t*-test. **g** Comet assay of murine colon organoid cells (each data point represents the mean of >80 cells) from three independent replicates shows more DNA damage in *pks*+ WT *E. coli*-infected cells, which is comparable to etoposide, the positive control. Data represent mean ± SD, *p* < 0.05, as calculated by one-way ANOVA and Tukey’s test. Source data are provided as a Source Data file.

To test whether the previously observed phenotypes of megalocytosis and DNA damage can be reproduced upon infection of human colon adenocarcinoma Caco-2 cells with the colibactin-producing (*pks*+) wild-type (WT) M1/5 *E. coli*, we infected this cell line for 3 h at multiplicity of infection (MOI) 20. To ensure the effects were colibactin-specific, we used the *E. coli ΔclbR* mutant strain^26^, which is defective for colibactin synthesis, as the negative control. Cells were immunostained for γH2AX, an early response to DNA damage required for the recruitment of DNA damage repair proteins. We observed γH2AX-positive cells, as well as megalocytosis and multinucleated cells in cultures infected with *pks*+ WT *E. coli*, while cultures infected with the mutant strain exhibited significantly less DNA damage (Fig. 1a). We next examined whether similar phenotypic changes are also observed after infection of primary cells. As it is difficult to image megalocytosis and multinucleated cells in organoids, we utilized mucosoids^27^, polarized epithelial monolayers grown in air–liquid interface^28^. For this, mouse primary colon cells were first grown as conventional 3D organoids^29^, then sheared into single cells and seeded on collagen-coated polycarbonate filter inserts. Cultures in air–liquid interface form highly polarized monolayers, with secreted mucus accumulating on the apical side, similar to the epithelium in vivo (Fig. 1b). Immunolabeling of cultures shows that the cells express β-catenin and MUC2, the typical mucin of the colon (Fig. 1c, top). They became fully polarized, as indicated by basolateral E-cadherin staining and the presence of eccentric nuclei, and focally expressed Ki67, a marker of proliferating cells (Fig. 1c, bottom). After infection at MOI 5 for 3 h, we detected γH2AX-positive cells, megalocytosis, and multinucleated cells, similar to the phenotypes seen in Caco-2 cells (Fig. 1d). These phenotypes were not seen after infection with the *ΔclbR* mutant. Thus, primary cells show a similar phenotypic response to colibactin as previously observed in cell lines.

We also infected murine colon organoids with *pks*+ *E. coli* at MOI 5 for 3 h. Organoids were first broken up, so that both the basal and apical sides were exposed to the bacteria. Whole-mount immunofluorescence labeling was carried out 3 days later, after organoids had re-formed. Again, we observed γH2AX-positive cells only after infection with the *pks*+ WT *E. coli* strain, indicating that this system is suitable for studying the effects of colibactin on primary cells (Fig. 1e). To confirm that the effects observed in the context of primary cell infection were not due to differences in infectivity between WT and *ΔclbR* mutant *E. coli*, we performed colony-forming unit (CFU) analysis upon infection of organoids, confirming equal colonization rate (Fig. 1f).

 To further confirm the presence of DSBs, we measured damaged DNA in murine colon organoid cells by neutral comet assay. The amount of DNA damage caused by infection with *pks*+ WT *E. coli* was similar to that induced by treatment with etoposide, used as a positive control. The *ΔclbR* mutant caused only minimal DNA damage compared to the non-infected condition (Fig. 1g). Infection of human colon organoids also resulted in γH2AX-positive cells and caused significantly more DNA damage in neutral comet assay only in the *pks*+ WT *E. coli*-infected condition (Supplementary Fig. 1a, b). Furthermore, upon infection with *pks*+ WT *E. coli*, human colon organoids showed nuclear expression of the DSB repair proteins, phospho-checkpoint kinase 2 (Chk2) (Supplementary Fig. 2a), and p53-binding protein 1 (53BP1) (Supplementary Fig. 2b), indicating that DSB repair is activated in response to colibactin. We therefore conclude that primary cells show DNA damage, megalocytosis, formation of multinucleated cells, and expression of DSB repair proteins upon exposure to *pks*+ *E. coli*, which is dependent on the synthesis of colibactin.

### Wnt-independent growth of organoids after infection with *pks*+ *E. coli*

Although DSBs are known to cause apoptosis or cellular senescence, immortalized Chinese hamster ovary cells have been shown to continue to proliferate in the presence of DNA damage after infection with *pks*+ *E. coli*^16^. We wanted to explore the response of non-immortalized primary cells equipped with normal DNA repair mechanisms. Primary polarized epithelial monolayers were infected with *pks*+ *E. coli* and immunolabeled for γH2AX and Ki67. After 3 h of infection, several cells were double-positive for γH2AX and Ki67, indicating that they proliferated despite DNA damage. In contrast, monolayers that were treated with cisplatin, a chemotherapeutic agent known to cross-link DNA and form cytotoxic adducts similar to colibactin^30^, contained no double-positive cells (Supplementary Fig. 3a). Immunofluorescence also showed a lower expression of γH2AX for cisplatin-treated cells, which was compatible with the neutral comet assay results (Supplementary Fig. 3b).

**Fig. 2:**
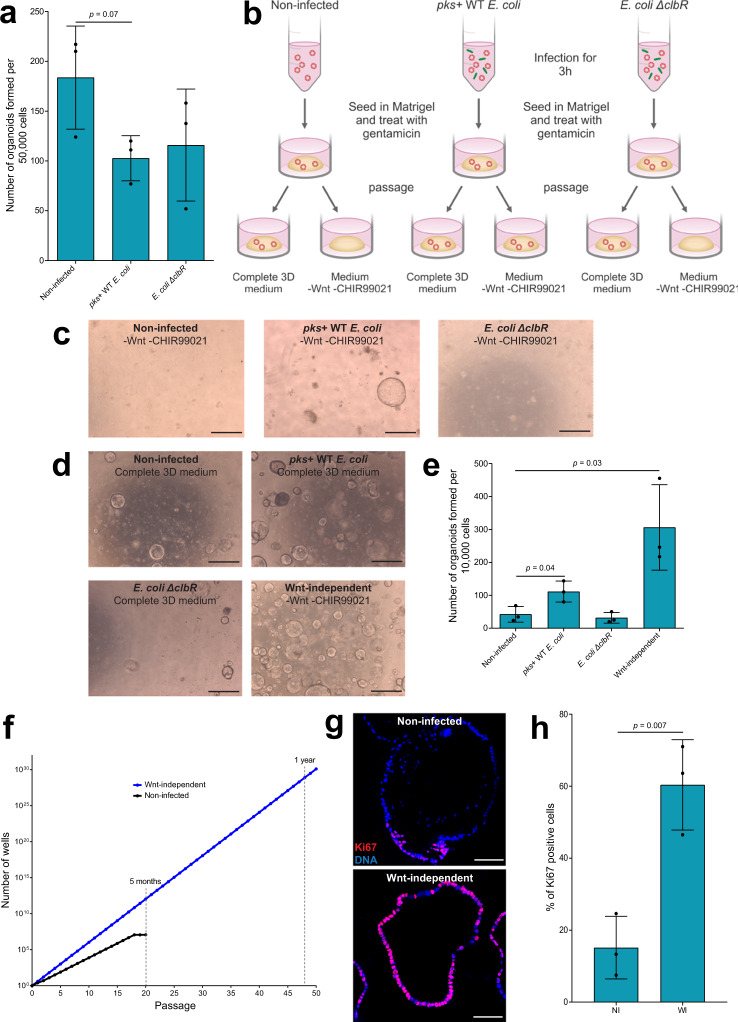
Wnt-independent growth of organoids after infection with *pks*+ *E. coli*. **a** Organoids formed after seeding 50,000 non-infected, *pks*+ WT *E. coli*-infected, and *E. coli ΔclbR*-infected cells from three independent replicates. Data represent mean ± SD, *p* < 0.05, as calculated by two-sided unpaired Student’s *t*-test. **b** Scheme describing the infection of organoids. **c** Organoids grow without the addition of Wnt and CHIR99021 to the medium for the *pks*+ WT *E. coli-*infected condition, but not for the non-infected or *E. coli ΔclbR*-infected conditions. Image representative of four independent replicates. Scale bars: 1 mm. **d** Organoids formed after seeding 10,000 cells in each condition. Image representative of three independent replicates. Scale bars: 1 mm. **e** Quantification of the number of organoids formed after seeding 10,000 cells in each condition from three replicates. Data represent mean ± SD, *p* < 0.05, as calculated by two-sided unpaired Student’s *t*-test. **f** Wnt-independent organoids have a higher splitting ratio and grow indefinitely when compared to non-infected organoids. Graph representative of four independent replicates. **g** Immunofluorescence staining for Ki67 (red) in non-infected organoids and Wnt-independent organoids. Hoechst (blue) stains for DNA. Image representative of three independent replicates. Scale bars: 100 μm. **h** Quantification of the percentage of Ki67-positive cells from three replicates. Data represent mean ± SD, *p* < 0.05, as calculated by two-sided unpaired Student’s *t*-test. NI = non-infected and WI = Wnt-independent. Source data are provided as a Source Data file.

Cell proliferation in the presence of incompletely repaired DNA damage can cause somatic mutations, which could potentially lead to independence from niche factors. Such niche escape is a hallmark of cancer organoids^2,3^. To assess their organoid-forming capacity, we infected organoids at MOI 5 for 3 h, re-seeded them in Matrigel and treated them with gentamicin to kill the bacteria. To determine the extent of cell death, we performed an organoid-forming assay where 50,000 cells were seeded in each condition. We observed that the organoid-forming efficiency for *pks*+ WT *E. coli*-infected organoids was reduced by about 40%, but there was no difference between WT and *ΔclbR* mutant *E. coli*-infected conditions (Fig. 2a). As the earliest and most frequently observed mutations in CRC are ones that lead to constitutive Wnt pathway activation, at the next passage we re-seeded the organoids into complete 3D medium, which contains both Wnt and the Wnt agonist CHIR99021, as well as into medium lacking these two factors (Fig. 2b). We noticed that non-infected and *ΔclbR* mutant-infected organoids failed to re-grow in Wnt-free medium, while in the *pks*+ WT *E. coli*-infected condition a few organoids did re-form (Fig. 2c). These Wnt-independent organoids could be further passaged and expanded. Thus, a short exposure of primary cells to colibactin-producing *E. coli* was sufficient to cause somatic adaptation that led to the generation of Wnt-independent clones.

We also performed experiments with *E. coli* Nissle 1917, which is widely used as a probiotic and known to harbor the *pks* island. Neutral comet assay demonstrated that Nissle 1917 caused fewer DSBs compared to the M1/5 strain, but more than was seen in the non-infected and the *E. coli* Nissle 1917 *ΔclbB* mutant-infected conditions (Supplementary Fig. 4a). Furthermore, we analyzed the cross-linking capacity of these two strains. WT bacteria were added to 1.5 µg of pfdA8 plasmid DNA at CFUs ranging from 1*10^6^ to 10*10^6^. A single CFU of 120*10^6^ was added for the mutant strains. A band at 3.4 kb indicates the presence of cross-linked DNA. Although both strains are commensal *E. coli*, the M1/5 strain showed a higher amount of cross-linked DNA compared to the Nissle 1917 strain. The absence of a band at 3.4 kb for the mutant strains indicated that they did not cross-link DNA (Supplementary Fig. 4b). Upon infection of murine colon organoids, no Wnt-independent organoids were observed in the *E. coli* Nissle 1917-infected condition, whereas the *E. coli* M1/5 strain once again generated Wnt-independent organoids. Additionally, treatment with etoposide or cisplatin also did not generate Wnt-independent organoids (Supplementary Fig. 4c). Therefore, the genotoxicity varies between strains of *pks*+ *E. coli* and also as compared to other DNA damaging agents.

### Wnt-independent organoids exhibit increased organoid formation capacity, higher proliferation, and longevity

To characterize the consequences of infection on organoid growth, we measured the organoid-forming capacity of non-infected, *pks*+ WT *E. coli*-infected and *ΔclbR* mutant-infected organoids as well as the Wnt-independent organoids generated in the previous experiment. After dissociation, 10,000 single cells were re-seeded from each condition, and the number of organoids formed was quantified. Cells that were obtained from the WT *pks*+ *E. coli*-infected condition had a higher organoid-forming capacity compared to those that were non-infected or infected with the *ΔclbR* mutant. However, the highest capacity was observed for cells derived from the Wnt-independent organoids (Fig. 2d, e). The Wnt-independent organoids were also found to grow for a longer period than non-infected organoids, which could only be maintained for about 20 passages. All biological replicates of Wnt-independent organoids were maintained in culture for more than 1 year, with one replicate maintained for over 2.5 years, and none showed any sign of slowed growth when the culture was discontinued. The passaging ratio was also higher for the Wnt-independent organoids, reflecting their increased growth capacity (Fig. 2f). Accordingly, immunolabeling of organoids 7 days after passaging showed that Wnt-independent organoids had a higher percentage of Ki67-positive cells when compared to non-infected organoids (Fig. 2g and h). In summary, Wnt-independent colon organoids generated by the action of colibactin had increased organoid-forming capacity, an increased number of proliferating cells, and potentially unlimited expansion capacity in culture.

### Wnt-independent organoids show upregulated Wnt/β-catenin signaling and downregulation of differentiation genes

**Fig. 3:**
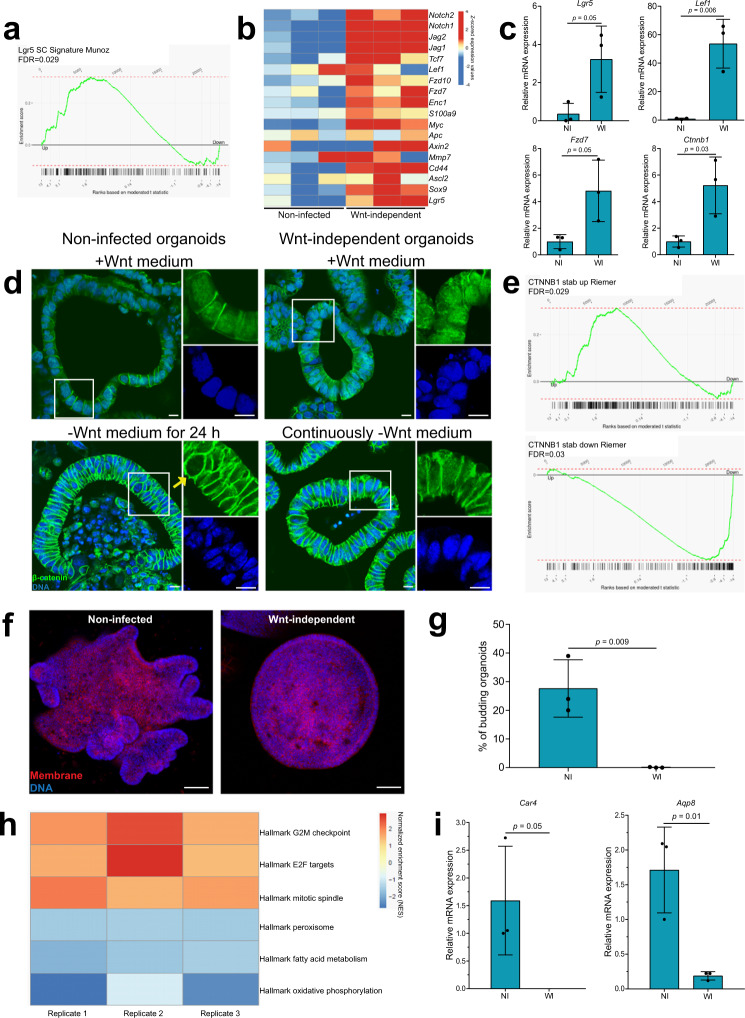
Wnt-independent organoids show upregulated Wnt/β-catenin signaling and downregulation of differentiation genes. **a** Global comparison using gene set enrichment analysis between intestinal stem cell genes^31^, and RNA sequencing results from three replicates. Genes are ordered according to their differential expression in Wnt-independent vs. non-infected samples from left to right. Intestinal stem cell genes are marked by black bars below the plot. Plot line shows the running enrichment score. **b** Wnt-independent organoids have a higher expression of Wnt target genes when compared to respective non-infected conditions. Data are mean-centered and averaged expression values for each replicate (*n* = 3) and condition. **c** RT-qPCR data showing expression of *Lgr5*, *Lef1*, *Fzd7*, and β-catenin (*Ctnnb1*) relative to *Gapdh* from three independent replicates. Data represent mean ± SD, *p* < 0.05, as calculated by two-sided unpaired Student’s *t*-test. NI = non-infected and WI = Wnt-independent. **d** Immunostaining for β-catenin (green) in non-infected and Wnt-independent organoids. Upon removal of Wnt from the medium for 24 h, non-infected organoids show the absence of nuclear β-catenin (marked by the yellow arrow). DAPI (blue) stains for DNA. Image representative of three independent replicates. Scale bars: 10 μm. **e** Global comparison using gene set enrichment analysis between genes upregulated and downregulated during activated β-catenin signaling^32^, and RNA sequencing results from three replicates. Genes are ordered according to their differential expression in Wnt-independent vs. non-infected samples from left to right. Genes are marked by black bars below the plot. Plot line shows the running enrichment score. **f** Immunofluorescence image of a single organoid on day 7 after seeding. Image representative of three independent replicates. Scale bars: 100 μm. **g** Bar graph of the percentage of differentiated organoids from three replicates on day 7 after seeding. >90 organoids counted per condition for each replicate. Data represent mean ± SD, *p* < 0.05, as calculated by two-sided unpaired Student’s *t*-test. NI = non-infected and WI = Wnt-independent. **h** Hallmark genes associated with mitosis (Hallmark G2M checkpoint, Hallmark E2F targets, and Hallmark mitotic spindle gene sets) are enriched among upregulated genes, and hallmark genes associated with differentiation (Hallmark peroxisome, Hallmark fatty acid metabolism, and Hallmark oxidative phosphorylation gene sets) are enriched among downregulated genes in all three replicates of Wnt-independent organoids when compared to non-infected organoids. Normalized enrichment score (NES) for a given gene set is the enrichment score normalized to mean of the enrichment scores for all data set permutations. Results are significant (FDR < 20%), except for Hallmark oxidative phosphorylation of replicate 2. **i** RT-qPCR data showing expression of *Car4* and *Aqp8* relative to *Gapdh* from three independent replicates. Data represent mean ± SD, *p* < 0.05, as calculated by two-sided unpaired Student’s *t*-test. NI = non-infected and WI = Wnt-independent. Source data are provided as a Source Data file.

Next, we determined if stem cell-associated pathways were upregulated in Wnt-independent organoids. We performed RNA sequencing of non-infected and Wnt-independent organoids. All cultures were grown in a complete 3D medium for 5 days, followed by growth in medium without Wnt and CHIR99021 for 3 days. GSEA revealed a significant enrichment of Lgr5 signature genes^31^ in Wnt-independent organoids when compared to non-infected organoids (Fig. 3a). All three replicates of Wnt-independent organoids expressed higher levels of Wnt target genes when compared to the respective non-infected cultures (Fig. 3b). We performed RT-qPCR for *Lgr5* (the classic intestinal stem cell marker), *Lef-1*, *Fzd7*, and β-catenin (*Ctnnb1*) and found higher expression of these genes in Wnt-independent organoids (cultured in −Wnt medium) when compared to non-infected organoids (cultured in +Wnt medium) (Fig. 3c). Immunostaining for β-catenin showed the presence of nuclear β-catenin in Wnt-independent organoids in the presence or absence of Wnt, indicating active Wnt/β-catenin signaling. Non-infected organoids kept in the absence of Wnt for 24 h showed absence of nuclear β-catenin in several nuclei (Fig. 3d). Furthermore, gene sets implicated in Wnt signaling that are upregulated or downregulated due to stabilization of β-catenin in intestinal organoids^32^ showed a significant positive and negative enrichment, respectively, in Wnt-independent organoids when compared to non-infected organoids (Fig. 3e). The Wnt-independent organoids rely on endogenous Wnt production because treatment with a porcupine inhibitor (IWP-2) led to inhibition of their growth, which could be rescued by exogenous Wnt (Supplementary Fig. 5a). This indicated that these organoids showed a higher sensitivity to endogenous Wnt. We explored the expression pattern of selected genes encoding for Wnt receptors and found a higher expression of *Lrp6*, *Fzd7*, and *Fzd10*, which could be responsible for the increased sensitivity to endogenous Wnt signals (Fig. 3b, c and Supplementary Fig. 5b).

Upregulated Wnt signaling in intestinal organoids has previously been shown to cause cells to adopt a proliferative progenitor phenotype, which results in a shift from the asymmetric crypt-villus architecture to a spheroid, cyst-like morphology devoid of differentiated cell types^33^. Accordingly, we noticed that Wnt-independent organoids formed spheroid, cyst-like organoids, unlike the non-infected condition (Fig. 3f). To quantify this, all conditions were kept in complete 3D medium for 7 days, and any organoid showing a budding structure was scored as a differentiated organoid (Fig. 3g). Further, GSEA revealed an upregulation of gene sets implicated in proliferation and downregulation of gene sets involved in fatty acid metabolism, which are active in differentiated colonocytes (Fig. 3h). In addition, the enterocyte-specific differentiation genes carbonic anhydrase 4 (*Car4*) and aquaporin 8 (*Aqp8*) were downregulated in Wnt-independent organoids (Fig. 3i). Thus, infection-induced Wnt-independence of organoids was accompanied by a proliferative cell phenotype and impaired differentiation.

### Wnt-independent organoids have major chromosomal aberrations and high mutational load

**Fig. 4:**
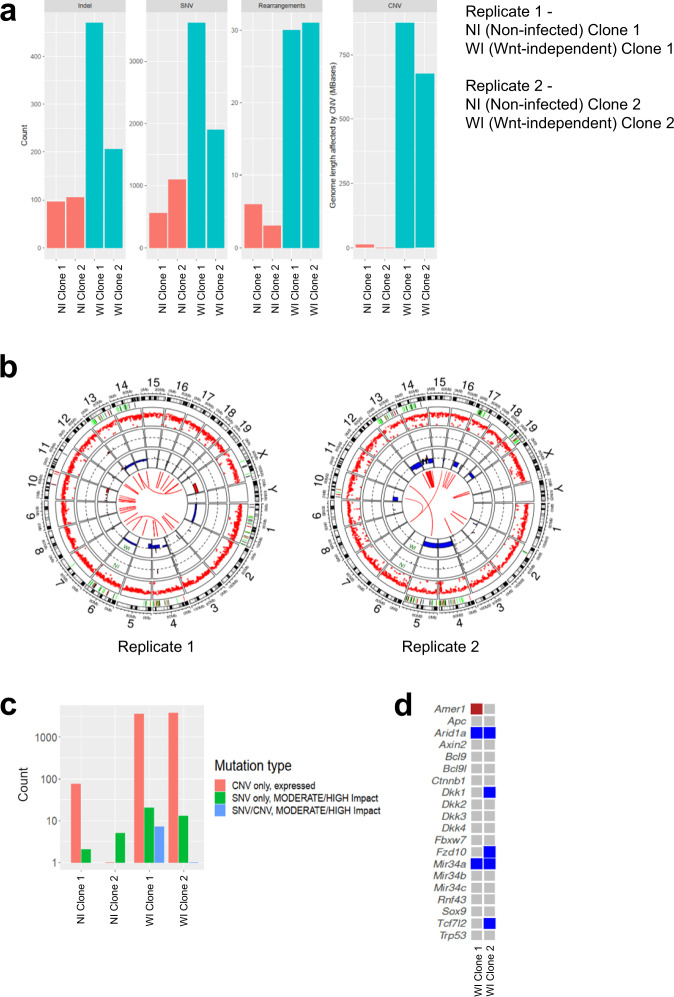
Wnt-independent organoids have major chromosomal aberrations and high mutational load. **a** The number of detected insertions and deletions (indels), somatic single nucleotide variants (SNV), and detected rearrangement breakpoints (Rearrangements) in Wnt-independent (WI) and non-infected (NI) organoids in two independent replicates from whole-genome sequencing (WGS). Genome length affected by copy number variations (CNV) for Wnt-independent (WI) and non-infected (NI) organoids in two independent replicates from WGS. Each condition was compared to the pool of non-infected organoids of that respective experiment. **b** Circos plots showing CNV, SNV, indels, structural variants, and mutated genes in the Wnt-independent organoids for replicates 1 and 2 from WGS. Each section represents chromosomes 1–19, chromosome X, and chromosome Y. The central part shows aberrant genomic fusions in the Wnt-independent condition. The two innermost circles (WI and NI) represent CNV in non-infected (NI) organoid clones and Wnt-independent (WI) organoid clones, respectively. The blue (genomic loss) and red (genomic gain) regions represent CNV. All losses of chromosomal regions are heterozygous. Each condition was compared to all the non-infected organoids of that respective replicate. A rainfall plot (red dots) shows the SNV and indel variations and the distances between them on the *y*-axis only for the Wnt-independent condition. The green, orange, and black lines depict positions of mutated Wnt pathway genes, known cancer driver genes, and p53 pathway genes, respectively, in the Wnt-independent condition. **c** The number of genes affected in each sample by CNV and/or SNV analyzed by WGS. For CNV, only genes expressed in non-infected controls were considered. For SNV, only mutations that change the amino acid sequence are shown. **d** Mutational status of genes of the Wnt pathway that are frequently affected in CRC, genes of the micro-RNA-34 family, and *Trp53*. Blue, genomic loss; red, genomic gain. Source data are provided as a Source Data file.

To determine whether the alterations induced by colibactin are linked to mutations, we generated clonal and pooled cultures from non-infected and Wnt-independent organoids and performed whole-genome (WGS) and whole-exome sequencing (WXS) on cells obtained from two replicates. We identified a higher number of clonal (allelic proportion >25%) single nucleotide variants (SNV), insertion and deletion (indel) mutations, and detected rearrangement breakpoints (rearrangements) in Wnt-independent organoids compared to non-infected controls. Approximately 875 million and 650 million base pairs were affected by copy number variations (CNV) in clones generated from Wnt-independent organoids from replicate 1 and replicate 2, respectively (Fig. 4a). However, many of the identified mutations were shared between clones from the same mouse (genomic losses on chromosomes 4, 5, and 13 in replicate 1 and chromosomes 2, 4, 5, 10, 13, 17, and 19 in replicate 2), indicating that they may have originated from the same mutated cell (Supplementary Fig. 6a). Thus, the Wnt-independent cells show features of CIN with CNV, including predominant losses of whole chromosomes or chromosome arms (Fig. 4b), in agreement with chromosomal aberrations induced during mitosis^16^. Subclonal changes such as additional breaks, genomic gains, and genomic losses indicate ongoing instability, including chromosome 14 of replicate 2, which exhibited multiple breakpoints and CNV in close proximity with pure intrachromosomal relocations, which may be a sign of chromothripsis^34^. Over 3600 genes were found to be affected, mostly by CNV, but to a lesser extent by SNV or by both SNV and CNV. Copy-number imbalances affected about 3500 expressed protein-coding genes in each sample, while only about 10–20 genes showed protein changes due to single nucleotide substitutions or small indels (Fig. 4c). Among them were genes known to be involved in Wnt signaling, in the p53 pathway, and known cancer drivers (Fig. 4d). We did not identify CNV or known driver mutations in the mouse homologs of the key Wnt-signaling genes *Apc* or *Ctnnb1*. Instead, Wnt-independent clones showed copy number changes in a number of additional genes frequently mutated across cancers^35^ or specifically in colorectal carcinoma^4^ (Supplementary Fig. 6b). Both replicates had a heterozygous loss of AT-rich interactive domain-containing protein 1 A (*Arid1a*), which is mutated in several types of cancer^36^. *Arid1a* has been shown to act as a tumor suppressor in the mouse colon by enhancer-mediated gene regulation^37^, and in ovarian cancer *ARID1A* mutation has been shown to functionally inactivate *TP53*^38^. In both replicates of exome sequencing, there was a heterozygous loss of the micro-RNA-34a (*miR-34a*) gene. *miR-34a* expression is induced by p53 and is known to play important roles in the downregulation of the Wnt pathway and in tumor suppression^39,40^. In one of our replicates, there was a heterozygous loss of cyclin-dependent kinase inhibitor 1a (*Cdkn1a/p21*) gene, while the other one showed a loss of cyclin-dependent kinase inhibitor 1b (*Cdkn1b*), both of which are involved in cell cycle arrest. *Cdkn1a* is a target of p53 and acts downstream to inhibit cellular proliferation. In both replicates, there was also a heterozygous loss of cyclin-dependent kinase inhibitor 2a (*Cdkn2a*), known to act upstream of p53. One replicate had gain-of-function of cyclin-dependent kinase 2 (*Cdk2*) and cyclin-dependent kinase 4 (*Cdk4*), genes involved in cell cycle progression that are negatively regulated by p53. The Wnt and p53 pathways are complex regulatory networks, therefore it is not unlikely that a mutation or a combination of mutations induced by colibactin disrupted these critical pathways, which are important for cellular proliferation and tumorigenesis.

A mutational signature of colibactin action on DNA has recently been identified^23,24^. We tested if we could identify hallmarks of this signature in our Wnt-independent clones. We neither identified the previously described increase in T > N substitutions at ATA, ATT, and TTT^24^ (Supplementary Fig. 6c) nor a relevant increase in small indels at A:T homopolymers (Supplementary Fig. 6d). We also did not observe an enrichment of structural variant breakpoints at the reported AAWWTT motif^23^ (Supplementary Fig. 6e). Based on previous data^24^, only a small number of colibactin-associated SNV and small indels are expected in our experimental setting (i.e., about 4 SNV and 1 indel after 3 h infection compared to about 500 additional single base substitutions and 150 indels after 5 repetitions of 3 days of infection^24^, assuming a uniform rate of mutations over time). On the other hand, chromosomal aberrations have been observed 24 h after a 4 h exposure to colibactin-producing *E. coli*^16^. Indeed, our data indicate that colibactin-induced cross-links might lead to genomic instability even after short contact, without evidence of a mutational signature, which most likely requires long-term or repetitive exposure to colibactin.

These sequencing results, therefore, show the genomic lesions in primary colon cells resulting from a single 3 h infection with *pks*+ *E. coli*. We conclude that Wnt-independent organoids show hallmarks of CIN and an increased mutational load affecting large numbers of genes, some of which are tightly linked to the p53 signaling pathway, which individually or in combination could have led to the development of niche independence.

### The Trp53/miR-34 axis is disrupted in the Wnt-independent organoids

**Fig. 5:**
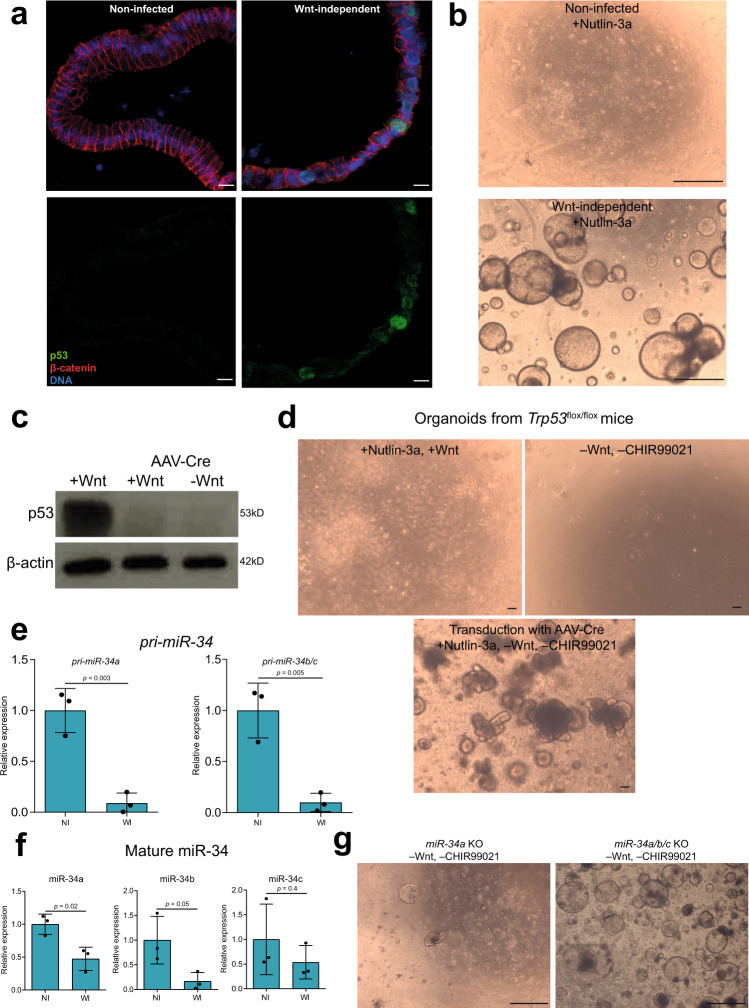
The Trp53/miR-34 axis is disrupted in the Wnt-independent organoids. **a** Organoids immunostained for p53 (green) and β-catenin (red). DAPI (blue) was used to stain for DNA. Image representative of three independent replicates. Scale bars: 10 μm. **b** Wnt-independent organoids grow in the presence of Nutlin-3a in the medium. Image representative of three independent replicates. Scale bars: 1 mm. **c** Western blot to verify knockout of *Trp53* in organoids after transduction with AAV-Cre. Image representative of two independent replicates. **d** Organoids from *Trp53*^flox/flox^ mice grow in the presence of Nutlin-3a and absence of Wnt and CHIR99021 only when transduced with AAV-Cre. Image representative of two independent replicates. Scale bars: 100 μm. **e** RT-qPCR data showing expression of *pri-miR-34* relative to TUBB from three independent replicates. Data represent mean ± SD, *p* < 0.05, as calculated by two-sided unpaired Student’s *t*-test. NI = non-infected and WI = Wnt-independent. **f** RT-qPCR data showing expression of mature miR-34 relative to U6 from three independent replicates. Data represent mean ± SD, *p* < 0.05, as calculated by two-sided unpaired Student’s *t*-test. NI = non-infected and WI = Wnt-independent. **g** Organoids from *miR-34a* KO mice do not grow in the absence of Wnt and CHIR99021, whereas organoids from *miR-34a/b/c* KO mice do grow in the absence of Wnt and CHIR99021. Image representative of two independent replicates. Scale bars: 1 mm. Source data are provided as a Source Data file.

As we noticed that several genes involved in p53 signaling were affected in the Wnt-independent organoids upon exposure to *pks*+ WT *E. coli*, we immunolabeled the organoids for p53 and observed the presence of nuclear p53 in several cells (Fig. 5a). We next checked the sensitivity of Wnt-independent organoids to Nutlin-3a, which activates the p53 pathway and acts as an apoptosis inducer. While non-infected organoids did not grow when Nutlin-3a was added to the medium, the Wnt-independent organoids continued to grow (Fig. 5b), indicating a defect downstream of p53.

To examine if there is a connection between Wnt-independence and disruption of the p53 pathway, we first tested whether loss of the *Trp53* gene results in organoids that grow in the absence of Wnt. For this, we generated organoids from the colon of *Trp53*^flox/flox^ mice. The *Trp53* gene was floxed out by the transduction of organoids with an adeno-associated virus encoding the Cre recombinase (AAV-Cre). To select cells with a successful knockout, the organoids were grown in the presence of Nutlin-3a. The depletion of p53 was further verified by western blot analysis. Non-transduced organoids were grown for 6 days and then kept in the presence of Nutlin-3a for 2 days to stabilize the p53 protein, while transduced organoids were cultured with Nutlin-3a for 8 days, confirming the loss of *Trp53* (Fig. 5c). The *Trp53* KO organoids continued to grow when Wnt and CHIR99021 were withdrawn from the medium, while non-transduced organoids grew neither in the presence of Nutlin-3a nor in the absence of Wnt (Fig. 5d). Therefore, loss of *Trp53* is sufficient to confer independence from extrinsic Wnt signals.

As we did not detect a mutation in the *Trp53* gene in the sequencing results, and as p53 was found in the nuclei, we hypothesized that Wnt-independence could be driven by an altered effector downstream of p53, and investigated if the loss of the *miR-34a* gene could have led to extrinsic Wnt-independence in the organoids. *miR-34a* is expressed at higher levels than *miR-34b/c*, with the exception of the lung, in which *miR-34b/c* is highly expressed^41^. RT-qPCR for *pri-miR-34* showed downregulation of *pri-miR-34a* and *pri-miR-34b/c* in Wnt-independent organoids when compared to non-infected organoids (Fig. 5e). Lower expression of mature miR-34a and miR-34b, but not miR-34c, was observed in Wnt-independent organoids (Fig. 5f). It has previously been shown that some epithelial-mesenchymal transition (EMT)-inducing transcription factors can bind to E-boxes of *miR-34a* and *miR-34b/c* promoters, thereby repressing *miR-34a* and *miR-34b/c* expression^42^. GSEA of the Wnt-independent organoids also showed an enrichment of EMT-related genes (Supplementary Fig. 7a).

To explore the function of miR-34, we generated colon organoids from *miR-34a*, *miR-34b/c*, and *mir-34a/b/c* KO mice, and found an increased expression of *Lgr5* and β-catenin (*Ctnnb1*) in *miR-34a* KO organoids, which was even more pronounced in *miR-34a/b/c* KO mouse-derived organoids. *miR-34a* KO and *miR-34a/b/c* KO organoids also showed increased expression of *Lrp6* and *Fzd7*, similar to the Wnt-independent organoids generated upon infection (Supplementary Fig. 7b). Furthermore, we observed that colon organoids generated from *miR-34a/b/c* KO mice were able to grow in the absence of Wnt and CHIR99021, whereas depletion of *miR-34a* alone was insufficient to maintain Wnt-independent long-term cultures (Fig. 5g). Thus, we conclude that the heterozygous loss of the *miR-34a* gene and the downregulation of miR-34a/b expression upon infection could together account for the observed phenotypic changes in colon organoids.

## Discussion

We demonstrate here the transformation of normal, healthy primary epithelial cells to a premalignant state upon short-term exposure in vitro to a bacterium that is a frequent constituent of the human intestinal microbiota. Specifically, infection of colon organoid cells with colibactin-producing *pks*+ *E. coli* led to the generation of mutant organoids that grew independently of Wnt. Aberrant Wnt signaling is observed in over 94% of CRC cases^4^. Also, niche factors have been shown to become dispensable for cancer organoids^2,3^. By selecting for Wnt-independence, we enriched for organoids that were mutated after exposure to *pks*+ *E. coli* out of a pool of non-affected or differently mutated cells. Importantly, to capture these clones, short-term infection with colibactin-producing *E. coli* was sufficient, and no Wnt-independent organoids were generated by infecting with an isogenic mutant defective for colibactin synthesis. This observation suggests a ‘hit-and-run’ mechanism for the transformation of normal primary cells to Wnt-independence upon contact with *pks*+ *E. coli*.

The Wnt-independent organoids generated upon infection exhibited accelerated growth, increased organoid formation, and prolonged expandability in culture. Additional signs of transformation included increased expression of Wnt target genes, loss of differentiation markers, and metabolic resemblance of these organoids to undifferentiated cells. Our extensive sequence analysis of Wnt-independent organoids generated by independently infected colon organoid lines revealed a plethora of mutations but none of them directly affected classical Wnt-signaling genes, such as *Apc*, which is often mutated in human colon cancer^4^. Nevertheless, the Wnt pathway was mostly affected indirectly by other mutational changes, e.g., in regulatory genes also frequently seen in human CRC. Likewise, *miR-34* members that exhibit defects in a subset of human cancers were heterozygously deleted and found to be downregulated in both replicates. miR-34a is known to be activated by p53 and downregulates Wnt signaling^39^. miR-34a was found to be silenced in approximately 75% and miR-34b/c in approximately 99% of sporadic CRC tumor samples^43,44^. In *Apc*^Min/+^ mice, miR-34a/b/c have been shown to suppress adenoma formation^45^. Ectopic expression of miR-34 induces cell cycle arrest in primary and tumor-derived cell lines^46^. miR-34a is also known to contribute to p53-mediated apoptosis and EMT suppression^42,47^. Moreover, *miR-34a* loss suppresses asymmetric division and increases Lgr5^+^ intestinal stem cell proliferation under inflammatory stress^48,49^. Interestingly, our infection-transformed organoids exhibited increased nuclear localization of p53 and resistance to Nutlin-3a. Heterozygouos loss of *miR34-a* and downregulation of miR-34a/b expression in the Wnt-independent organoids could potentially contribute to Nutlin-3a resistance. Further, our results showed that *Trp53* KO and *miR-34a/b/c* KO colon organoids can grow Wnt-independently, in concordance with previous work showing a functional overlap between these pathways^39^. *TP53* mutations are known to occur early in a subset of CRC, such as colitis-associated cancer^7^. Given the diversity of mutational patterns conferring Wnt-independence, considered as the hallmark of colon cancer initiation, it seems plausible that mutations other than the *APC* mutations frequently seen in advanced stages of cancer may arise at very early stages in the sequence of transforming events, yet have escaped attention. Mutations affecting *miR-34a*, as generated here by *pks*+ *E. coli*, may be such an example. Therefore, our data strongly suggest that disruption of the p53/miR-34 axis, along with additional mutations that occurred synergistically, resulted in the Wnt-independent phenotype in colon organoids. Such mutant cells displaying increased pro-survival signaling may possess a competitive growth advantage over normal cells and grow out to premalignant lesions, including polyps, in affected patients.

The mutational changes in the Wnt-independent organoids included SNV, but mainly CNV. Surprisingly, in these short-term infected organoids we failed to identify the specific mutational signature that we and others have recently shown to be induced by colibactin^23,24^. Notably, these previous studies derived from different experimental settings that aimed for the induction of high DNA damage or mutational burdens, yet did not explore the transforming potential of colibactin. In contrast, here, we exposed primary colon cells to *pks*+ *E. coli* only for very short times, reminiscent of the conditions in the dynamic process of crypt regeneration in the colon. This short exposure to colibactin sufficed to transform cells, suggesting that mutational changes distinct from the accretion of the recently published colibactin signature were involved.

Obviously, the characteristic colibactin signature is the result of an intricate damage and repair process ensuring minimal localized damage. Any failures to properly resolve the colibactin-induced cross-links, however, would lead to rather crude types of damage, such as severe chromosomal aberrations, in case mitosis proceeds^50^. This could explain the occurrence of multinucleated cells and aneuploidy as observed here, consistent with the occurrence of CIN, previously seen in intestinal epithelial cells after exposure to *pks*+ *E. coli*^16^. Resulting CNV have been identified as a predominant characteristic of the majority (~85%) of sporadic CRCs^51^, and are observed at early stages of the malignancy, facilitating cancer progression^52^. While in most cases mitotic stress due to aberrant or damaged chromosomes leads to mitotic arrest or cell death, some cells might evade this fate by mitotic slippage^53^. Accordingly, the occurrence of CIN and CNV following colibactin treatment is thought to be the result of inefficient local resolution of cross-links at the damaged sites, rendering cells prone to transformation, e.g., Wnt-independent growth.

The previous identification of the colibactin mutational signature^23,24^ in colon cancer genomes established a clear link between colibactin and the etiology of certain CRCs. The less-defined chromosomal aberrations resulting from improper repair of colibactin-induced damage, however, might play a prevailing role in cell transformation. Accordingly, the cancer-inducing potential of colibactin-producing bacteria might be higher than the frequency of the colibactin mutational signature suggests. Notably, the specific colibactin signature is found only in 2.4–10% of patients with CRC^23,24^, while colibactin-producing *E. coli* are found in 67% of CRC patients^17^.

We also observed variability in the genotoxicity between commensal *E. coli* strains. *E. coli* M1/5 is slightly more genotoxic than *E. coli* Nissle 1917, which is commonly used as a probiotic^54^. While we demonstrate that *E. coli* Nissle 1917 also causes DSBs and cross-links DNA, its damaging activity appears to be below the threshold, incapable of transforming cells to Wnt-independence in our assay. However, based on results in vitro, it is difficult to estimate the exact relative genotoxic activity in vivo, since it is subject to regulation by various factors, most prominently the availability of iron in the microenvironment of the infection at the pathogen–host cell interface^55,56^. While additional and as yet uncertain modulatory factors of colibactin expression, production, and delivery may play a role in terms of DNA damage-induction and cancer promotion, at this point it would appear prudent to view the use of colibactin-producing bacteria as probiotics with caution.

Taken together, our data demonstrate the transforming potential of *pks*+ *E. coli* in vitro and recapitulate the early stages of malignant transformation through infection of colon organoids. In particular, it highlights the relevance of CNV mutations leading to Wnt-independence as early drivers in colon cancer. While the colibactin mutational signature provides a clear link between colibactin action and colon carcinogenesis, colibactin’s transforming activity is likely higher than what can be estimated from the occurrence of that signature.

## Methods

### Bacterial strains, cell line, and mouse strains

The WT *E. coli* B2 *pks*+ M 1/5 human fecal isolate strain was used for the infections. The isogenic mutant strain used has *clbR* gene deleted in the *pks* island, which substantially hinders the ability of the bacterium to synthesize colibactin^26^. The WT and the mutant *E. coli ΔclbR* strain have a *rpsL* K42R mutation, which renders the strain streptomycin-resistant. For Supplementary Fig. 4, E*. coli* Nissle 1917 and *E. coli* Nissle 1917 *ΔclbB* strains were used. The cell line used was human colorectal adenocarcinoma cells Caco-2 (ATCC HTB-37). The cells were cultured in Dulbecco’s Modified Eagle Medium (DMEM, Gibco) +20% fetal calf serum (FCS, Biochrom). The mouse strains used were mTmG (JAX stock #007676)^57^, *Trp53*^flox/flox^ (Jackson stock #008462)^58^, *miR-34a* KO^45^, *miR-34b/c* KO^45^, and *miR-34a/b/c* KO^45^. All procedures involving animals were approved by the institutional and legal authorities at the Max Planck Institute for Infection Biology (Landesamt für Gesundheit und Soziales, Berlin). All animals were maintained in autoclaved microisolator cages and provided with sterile drinking water and chow ad libitum. Male 4–12 week old mice were used for this study.

### Generation of mutant *E. coli ΔclbR*

The deletion of *clbR* in *E. coli* strain M1/5 has been generated by lambda red-mediated recombineering^59^. A *npt* kanamycin resistance cassette was amplified from pKD4 (with primer pair GATAAGTTCAAAGAAAAAAACCCGTTATCTCTGCGTGAAAGACAAGTATTGCGCATGCTGTGTAGGCTGGAGCTGCTTC / ATATGAAAATCAATATTATCGACGGCTCAGAAGTGTCTAGATTATCCGTGGCGAT CATATGAATATCCTCCTTAGTTCC) and used for replacement of the *clbRA* genes in *E. coli* strain M1/5 (pKD46). The resulting mutant *E. coli* M1/5 Δ*clbRA* (pKD46) was then transformed with a PCR product amplified from pKD3-Δ*clbR1* (primer pair ATATGAAAATCAATATTATCGACGGCTCAGAAGTGTCTAGATTATCCGTGGCGATCATATGAATATCCTCCTTAGTTCC/ACCGTTGTATCGTAAATTCCTC). The PCR product included 132 bp of the *clbR* upstream region, the complete *clbA* gene, a *cat* cassette, an FRT site, and 55 nucleotides of the *clbA* downstream sequence. The PCR product was chromosomally inserted via recombination of two FRT site sequences upstream of *npt* and downstream of *cat*. The chromosomal *npt* and *cat* cassettes were then removed by transformation of this strain with pCP20 encoding the FLP recombinase^60^.

### Murine colon organoid culture, passaging, and cloning

The mice were sacrificed, and the colons isolated. The colons were cut longitudinally and cleaned with 1X phosphate-buffered saline (PBS) (Gibco), cut into small pieces, and the tissue fragments incubated in a cold chelating buffer (distilled water with 2 mM EDTA, 5.6 mM Na_2_HPO_4_, 8 mM KH_2_PO_4_, 96.2 mM NaCl, 1.6 mM KCl, 43.4 mM sucrose, 54.9 mM D-sorbitol, 0.5 mM DL-dithiothreitol) for 30 min at 4 °C on a roller mixer. The tube was vigorously shaken to resuspend tissue fragments and to isolate the colon crypts. The tissue fragments were allowed to settle by gravity, and the supernatant containing the crypts was removed and centrifuged at 300*g* for 5 min at 4 °C. The pellet was resuspended in Advanced DMEM/F-12 (Gibco) containing 10% heat-inactivated FCS and centrifuged at 300*g* for 5 min at 4 °C. The pellet was resuspended in 1 ml Advanced DMEM/F-12 containing 10% FCS and the number of crypts were counted using a hemocytometer. A total of 500 crypts were resuspended and seeded in a 40 µl Matrigel (Corning) drop in a 24-well plate. The Matrigel was polymerized at 37 °C for 10 min and then supplemented with 500 μl medium: Advanced DMEM/F-12 supplemented with 10 mM HEPES (Gibco), 1% Glutamax (Gibco), 50% Wnt-3A conditioned medium, 25% R-spondin-1 conditioned medium, 100 U/ml penicillin/streptomycin (Gibco), 1X N2 (Gibco), 1X B27 (Gibco), 1.25 mM N-acetylcysteine (Sigma), 50 ng/ml murine epidermal growth factor (mEGF) (Invitrogen), 100 ng/ml murine noggin (Peprotech), 0.5 μM A-83-01 (Sigma), 3 μM CHIR99021 (Stemgent), and 9 μM Y-27632 dihydrochloride (Sigma). The organoids were incubated at 37 °C, 5% CO_2_ in a humidified incubator. To the medium, 10 μg/ml metronidazole (Sigma) and 10 μg/ml ciprofloxacin (Bayer) were added additionally for the first 3 days. The medium was changed twice a week. Every 7 days, the organoids were passaged 1:2–1:5 by mechanically disrupting organoids and Matrigel with cold 1X PBS before transferring into a 15 ml falcon tube. After centrifugation at 300*g* for 5 min at 4 °C, the pellet was treated with TrypLE (Gibco) and incubated for 5 min at 37 °C, followed by dissociation using a flame-polished glass Pasteur pipette. The dissociated organoids were washed with 10 ml Advanced DMEM/F-12 containing 10% FCS and centrifuged at 300*g* for 5 min at 4 °C. The pellet was resuspended in 1 ml advanced DMEM/F-12 containing 10% FCS, and the number of cells counted using a hemocytometer. The required number of cells were resuspended and seeded per 40 μl Matrigel drop in a 24-well plate. The Matrigel was polymerized for 10 min at 37 °C. Each well was supplemented with 500 μl of medium and incubated at 37 °C, 5% CO_2_ in a humidified incubator. For generating clones, a single organoid was isolated and passaged to obtain the required number of cells. The concentration of cisplatin (Sigma) was 50 μM for monolayers and 10 μM for organoids. Etoposide (Sigma) was used at 50 μM concentration, IWP-2 (Sigma) was used at 5 μM concentration, and Nutlin-3a (Sigma) was used at 10 μM concentration.

### Human colon organoid culture

The human colon biopsies and tissue samples were received from surgeries performed at the Charité University Medicine, with prior approval of the ethics committee of the Charité University Medicine, Berlin (EA1/300/15) and informed consent was obtained from all donors. The biopsies were kept in ChillProtec medium (Biochrom) at 4 °C, and the isolation of crypts for organoid culture was carried out within 2–3 h after surgery. The tissue was washed with 1X PBS and cut into small pieces, and the tissue fragments were incubated in a 1X cold chelating buffer for 30 min at 4 °C on a roller mixer. The remaining steps were the same as for the murine colon organoid culture. The human colon organoid medium contained Advanced DMEM/F-12 supplemented with 10 mM HEPES (Gibco), 1% Glutamax (Gibco), 38 ng/ml surrogate Wnt (U-Protein Express BV), 25% R-spondin-1 conditioned medium, 100 U/ml penicillin/streptomycin (Gibco), 1X N2 (Gibco), 1X B27 (Gibco), 1.25 mM N-acetylcysteine (Sigma), 10 ng/ml human epidermal growth factor (hEGF) (Invitrogen), 100 ng/ml human noggin (Peprotech), 0.5 μM A-83-01 (Sigma), 10 mM nicotinamide (Sigma), 10 µM SB202190 (Sigma), 10 nM prostaglandin E2 (Tocris), and 9 μM Y-27632 dihydrochloride (Sigma). The organoids were incubated at 37 °C, 5% CO_2_ in a humidified incubator. For the first 3 days, 100 µg/ml Primocin (Invivogen) was additionally added to the medium. The medium was changed twice a week. The passaging protocol was the same as for the murine colon organoids.

### Murine primary colon epithelial monolayer culture

Half a million cells derived from murine colon organoids were resuspended in 200 µl of medium and seeded into bovine collagen type I-coated (Gibco, 1 mg/ml) polycarbonate cell culture inserts (Merck Millicell) placed in a 24-well plate. For monolayers, the medium used for organoids was supplemented with 10% FCS. Outside the cell culture insert, 600 μl medium was added, and the plate was placed at 37 °C, 5% CO_2_ in a humidified incubator. After 3 days, the medium from the cell culture inserts was discarded. The medium outside the cell culture insert was changed twice each week.

### In vitro infections

We determined that MOI of 20 and MOI of 5 was ideal for Caco-2 and primary cells, respectively, to obtain DNA damage after a 3 h infection. Higher MOIs caused very high or absolute cell death. Caco-2 cells were grown on collagen-coated MatTek glass-bottom dishes. For infection of Caco-2 cells, the medium was removed from 70% confluent cells. Infection medium (DMEM containing 10% FCS and 10 mM HEPES) containing log-phase *E. coli* at MOI 20 was added to the cells. The cells were then kept for 3 h in an incubator at 37 °C. To stop the infection, cell culture medium containing 200 μg/ml gentamicin (Sigma) was added, and cells were incubated at 37 °C overnight. The next day, the medium was changed to medium without gentamicin.

For infection of organoids, Matrigel was mechanically disrupted with infection medium and transferred to a 15 ml Falcon tube. The organoids were broken up by passing them through a flame-polished glass Pasteur pipette, so that both the basal and apical sides were exposed to the bacteria. The organoids were centrifuged at 300*g* for 5 min at 4 °C in a 1.5 ml tube and resuspended in 250 μl of infection medium. Log-phase *E. coli* at MOI 5 were added, and the tube incubated for 3 h at 37 °C. The organoids were centrifuged at 300*g* for 5 min at 4 °C and washed 2 times with advanced DMEM/F-12 containing 10% FCS. The pellet was resuspended in the required amount of Matrigel, and 40 μl drops added to each well in a 24-well plate. The Matrigel was polymerized by placing at 37 °C for 10 min. Each well was supplemented with 500 μl of medium containing 200 μg/ml gentamicin. The plate was then incubated at 37 °C. The next day, the medium was changed to medium without gentamicin.

Monolayers were infected by adding 250 μl of infection medium containing log-phase *E. coli* at MOI 5 to the cell culture inserts and incubating at 37 °C for 3 h.

For obtaining CFUs, after 3 h of infection, the organoids were washed with 1X PBS 5 times. The organoids were then treated with 0.2% saponin (Sigma) for 10 min at 37 °C to dissociate the attached bacteria. Serial dilutions were made and plated on LB agar plates, and colonies were counted the following day.

### Immunofluorescence

Caco-2 cells were grown on MatTek glass-bottom dishes and washed with PBS and fixed with 3.7% paraformaldehyde (Sigma) for 1 h. Organoids were mechanically disrupted and dissociated from Matrigel with cold PBS, transferred into a glass tube and allowed to settle by gravity. The organoids were then fixed with 3.7% paraformaldehyde for 1 h. Monolayers were washed with PBS and fixed in 3.7% paraformaldehyde for 1 h. They were first embedded in histogel (Thermo Scientific), followed by embedding in paraffin (Carl Roth). After fixation, the tissue pieces, organoids, and monolayers were dehydrated (1 h incubations in 70% EtOH, 80% EtOH, 90% EtOH, absolute EtOH, 100% isopropanol, twice in xylene) and paraffinized, cut into 5 μm sections with a microtome, and mounted on glass slides before dewaxing. For whole-mount staining of organoids, they were fixed directly in the Matrigel by adding 500 μl of 3.7% paraformaldehyde for half an hour at room temperature, then washed with 1X PBS before labeling. For whole-mount staining of monolayers, the monolayers were washed with PBS and fixed in 3.7% paraformaldehyde for 1 h, and then the filter was cut out and stained. The staining was performed with the antibodies and dyes listed in Supplementary Table 1. Primary antibodies were diluted in blocking solution (1% BSA, 2% FCS, and 0.1% Tween 20 in 1X PBS) and incubated overnight at 4 °C. Secondary antibodies were diluted in blocking solution, incubated for 45 min at room temperature for paraffin sections, and overnight at 4 °C for whole-mount. Mounting was done with Mowiol or Vectashield mounting medium (Vectorlabs, Inc.). Images were acquired with a Leica TCS SP-8 confocal microscope and processed using ImageJ 1.52.

### Western Immunoblotting

For the western blots, organoids were harvested in 2X Laemmli buffer, separated by 10% SDS-PAGE gels, transferred to a PVDF membrane (PerkinElmer), blocked in TBS buffer supplemented with 0.1% Tween 20 (Merck) and 5% milk (AppliChem), and probed against primary antibodies p53 DO-1 (Santa Cruz Biotechnology, SC-126, 1:500 dilution) and β-actin (Sigma-Aldrich, A5441, 1:10000 dilution) at 4 °C overnight. Membranes were probed with matching secondary horseradish-peroxidase-conjugated antibodies (Amersham, 1:3,000) and detected with ECL reagent (PerkinElmer). An uncropped scan image of the blot is provided in the Source Data file.

### Real-Time qPCR

Organoids were released from Matrigel with cold 1X PBS and pelleted by centrifugation (300*g* for 5 min at 4 °C), followed by RNA isolation using the phenol-chloroform RNA extraction method. For primary miRNA, total RNA was isolated using the miRNeasy mini kit (Qiagen), and for analysis, 1 μg of total RNA per sample was used to generate cDNA using the Verso cDNA synthesis kit (Thermo Fisher). qPCR was performed using a Power SYBR Green RNA-to-CT 1-Step Kit (Applied Biosystems). Reactions were performed in 25 μl containing 50–200 ng RNA, 10 μl SYBR Green mix, 0.16 μl RT mix, plus primers at 0.2 μM each. Program: 30 min at 48 °C; 10 min at 95 °C; followed by 40 cycles of 15 s at 95 °C/60 s at 60 °C. For analyses of mature miRNA expression, cDNA was generated, and qPCR was performed using the miRCURY LNA RT kit (Qiagen), the miRCURY LNA™ SYBR Green Master Mix (Exiqon), and the following commercially available primers: mmu-miR-34a (Qiagen, YP00204486), mmu-miR-34b (Qiagen, YP00204424), mmu-miR-34c (Qiagen, YP00205659), and the snRNA U6 (Exiqon, 203907). Primers used are listed in Supplementary Table 2. For each oligonucleotide pair and RNA sample, the reaction was performed in triplicate. The data was analyzed using the delta-Cq method. The expression levels of the target genes were normalized to the levels of glyceraldehyde-3-phosphate dehydrogenase (*Gapdh*) gene expression in each individual sample. For primary miRNA, the expression was normalized to TUBB, and for miRNA, the expression was normalized to U6.

### DNA cross-linking assay

This method was adapted from Xue et al.^61^. Here, 1 ml of overnight culture was centrifuged at 1600*g* for 3 min. The pellet was resuspended in 20 ml M9-CAP medium and shaken at 200 rpm for 2 h at 37 °C. M9-CAP medium contained M9 minimum media (Difco) supplemented with 0.4% glucose, 2 mM MgSO_4_, 0.1 mM CaCl_2_, 12.5 μg/ml chloramphenicol, and the following L-amino acid mass composition (5 g/l total): 3.6% Arg, 21.1% Glu, 2.7% His, 5.6% Ile, 8.4% Leu, 7.5% Lys, 4.6% Phe, 4.2% Thr, 1.1% Trp, 6.1% Tyr, 5% Val, 4% Asn, 4% Ala, 4% Met, 4% Gly, 4% Cys, and 4% Ser. At this time point, OD measured at 600 nm is 0.8 and there are 1 × 10^9^ CFUs/ml. Serially diluted bacteria were added to 1.5 µg of pfdA8 plasmid DNA containing 3 µl of 50 mM EDTA and incubated for 90 min at 37 °C. Cleanup of DNA was done using QIAquick PCR Purification Kit (Qiagen), and 300 ng of DNA samples were loaded in a 1% denaturing agarose gel (1% agarose, 5% 1 M NaCl, and 2% 50 mM EDTA water). The gel ran at 50 V for 2 h in running buffer (5% 1 M NaOH and 0.2% 0.5 M EDTA in water). The DNA was stained with SafeRed (Biotium), and the gel was imaged under UV light.

### Neutral comet assay

The neutral comet assay was performed following infection, etoposide (Sigma) treatment or cisplatin (Sigma) treatment of single cells derived from organoids using the kit from Trevigen (4250-050-K), following the manufacturer’s protocol. Images were acquired using a fluorescent microscope (Leica DMR). The percentage of DNA in the tail was quantified using the software CometScore (TriTek 2.0).

### Transduction

The organoids were dissociated with TrypLE and resuspended in 300 μl culture medium (without penicillin/streptomycin and N-acetylcysteine) containing AAV-Cre adenovirus particles and 8 μg/ml polybrene (Sigma). Cells were seeded in a 24-well plate coated with Matrigel. After overnight incubation, the medium was removed, and a second layer of Matrigel was added and polymerized as described above, before adding 500 μl culture medium. The culture was passaged after 7 days.

### RNA sequencing

RNA sequencing was performed at the Max Planck Genome Centre, Cologne, using Illumina HiSeq3000. PolyA enrichment was applied to all samples. For each sample of replicate 1, about 50 M single-end reads (150 bp) were generated. For replicates 2 and 3, about 20 M paired-end reads (2 × 150 bp) were generated per sample. Gene expression was quantified by Salmon^62^, using pseudo-alignment of unaligned reads against transcript sequences from Gencode M12 (www.gencode.org). Gene quantification data generated by Salmon were further processed in R using package DESeq2^63^ to determine differentially expressed genes between conditions and samples.

### DNA sequencing

The sequencing of non-infected and Wnt-independent organoids for both replicates was performed at passage 11 and passage 13 after infection, respectively. All conditions were compared to all the non-infected organoids of that respective replicate. For each condition, DNA was extracted from approximately 1 million cells.

The exome sequencing was performed at the Max Planck Genome Centre, Cologne, using Illumina HiSeq3000. Exome targets were captured using SureSelectXT Mouse All Exon kit (Agilent Technologies) and sequenced using a paired-end library protocol (2 × 150 bp) with a target coverage of 60-fold at 80% on-target rate. Reads were aligned against mouse genome version GRCm38 using BWA. Duplicate marking, base call recalibration, and realignment around indels were done using GATK v3.4^64^. Somatic SNPs and small indels were called against pooled non-infected conditions as germline control using Strelka v1.0.11^65^ and annotated using SnpEff v4.3k^66^. CNV were determined using CONTRA v2.08^67^.

WGS was performed at BGI Tech Solutions, Hong Kong, using BGISEQ-500 with a paired-end (2 × 100bp) library protocol and a target coverage of 30-fold. Reads were mapped to GRCm38 using BWA. CNV for WGS data were called using ControlFreeC v10.8^68^ and R package DNAcopy. Copy number segments with absolute log2 ratio larger than 0.15 were called as gains or losses. Structural variants were called using SvABA^69^. SNV and indels were called in a similar way as for exome variants using GATK preprocessing and variant calling by Strelka.

For both exome and whole-genome sequencing, we kept only SNV and indels with allelic proportions of at least 25% in order to remove subclonal variants arising during expansion after treatment.

All tertiary analysis was done using R^70^, including package circlize^71^.

### Gene set enrichment analysis (GSEA)

Gene sets were used from MSigDB v6.2 (collections H, C2, C3, C5, C6, and C7)^72,73^ after mapping genes from mouse to human genes using the HomoloGene database^74^. Additionally, lists of intestinal stem cell signature genes^31^ and genes upregulated and downregulated during activated β-catenin signaling^32^ were used. The GSEA^75^ was performed on genes pre-ranked by gene expression-based log2 fold change between non-infected and Wnt-independent organoids using the fgsea R package^76^ with 5000 permutations; R v.3.4 was used (https://cran.r-project.org/). For each experiment, *p* values from all gene sets were jointly adjusted for multiple testing using the method of Benjamini–Hochberg (false discovery rate)^77^.

### Statistical analysis

All graphs were prepared using GraphPad Prism version 8 software. Data are presented as mean ± SD (standard deviation), and *p* values were calculated by two-sided unpaired Student’s *t*-test. For comet assays, one-way ANOVA with respective post hoc analysis (Tukey’s test) was used to calculate *p* values. Data were considered significant if *p* < 0.05. Unless stated otherwise, figures represent three independent experiments.

### Reporting summary

Further information on research design is available in the Nature Research Reporting Summary linked to this article.

## Supplementary information

Supplementary information.

Reporting summary.

## Data Availability

All RNA sequencing data are available from Gene Expression Omnibus (GEO) with ID GSE140929. Whole-genome and exome sequencing data have been submitted to the European Nucleotide Archive (ENA) under accession PRJEB35529. MSigDB v6.2 is available from http://www.gsea-msigdb.org/gsea/downloads_archive.jsp. Homologene build 68 is available from https://ftp.ncbi.nih.gov/pub/HomoloGene/. Gencode M12 mouse gene models were obtained from ftp://ftp.ebi.ac.uk/pub/databases/gencode/Gencode_mouse/release_M12/. Other data supporting the findings of this study are available within the paper and its Supplementary Information files, or from the corresponding authors upon request. Source data are provided with this paper.

## Source data

Source data.

## Code availability

All code is available from https://github.com/MPIIB-Department-TFMeyer/Iftekhar_Colibactin. The DOI link is 10.5281/zenodo.4322729^78^.
