# Supplementary material for: Genomic aberrations after short-term exposure to colibactin-producing E. coli transform primary colon epithelial cells
Source: Nat Commun. 2021 Feb 12;12:1003. doi: 10.1038/s41467-021-21162-y (PMC7881031; doi:10.1038/s41467-021-21162-y)
Supplement: Supplementary file 1 — Supplementary information. [file 41467_2021_21162_MOESM1_ESM.pdf]

Supplementary Information

Genomic aberrations after short-term exposure to colibactin-producing *E. coli* transform primary colon epithelial cells

Iftekhar A et al.

Supplementary Figure 1:

a

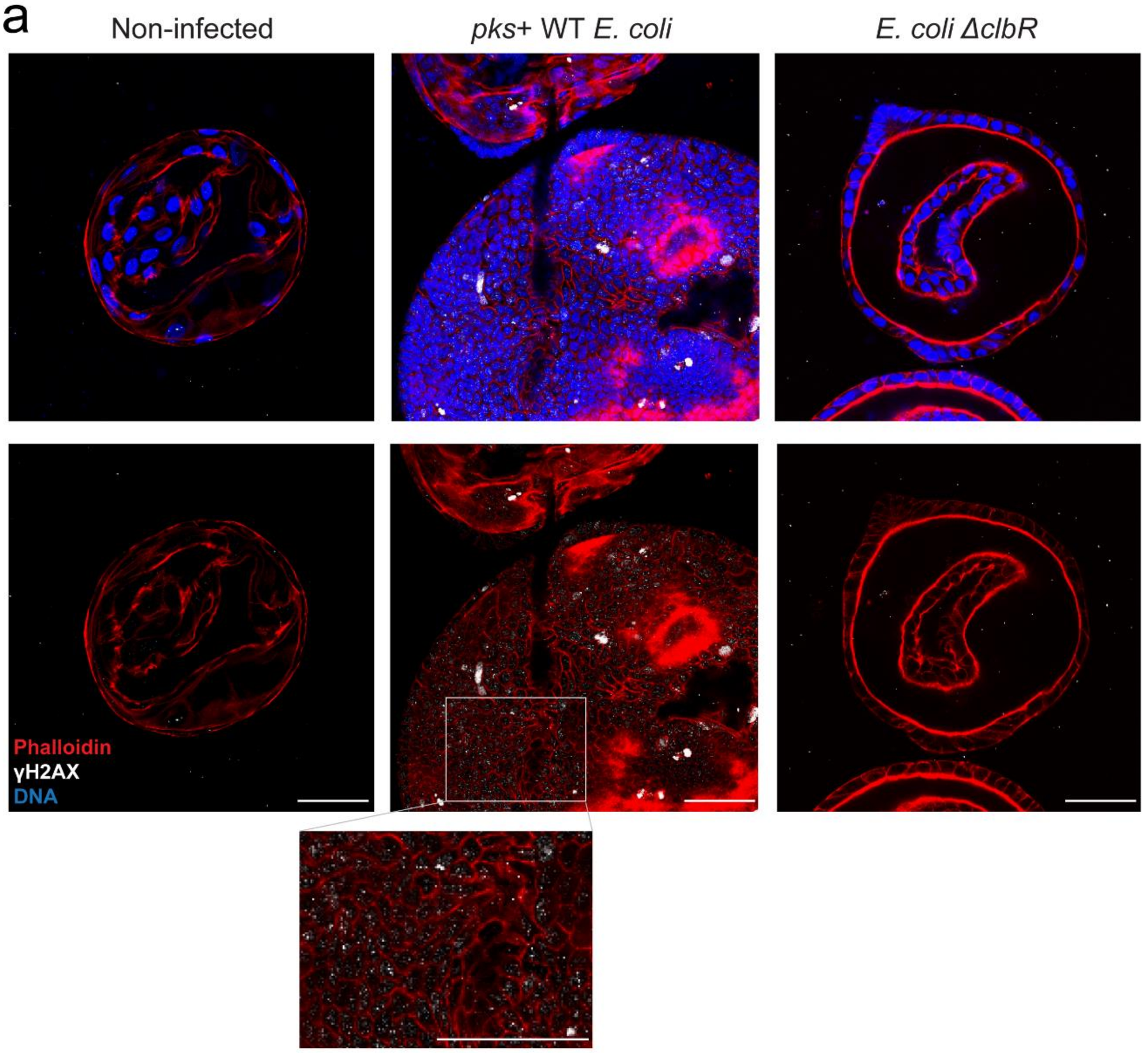

b

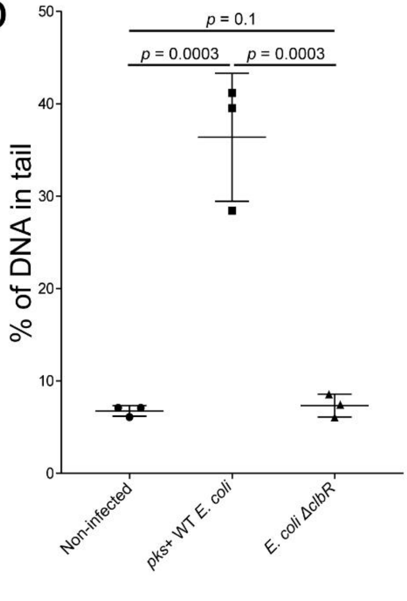

Supplementary Figure 1: *pks*+ *E. coli* induce DNA damage in human colon organoids

a. Human colon organoids were immunolabelled for γH2AX (white). Phalloidin (red) stains for actin filaments and Hoechst (blue) stains for DNA. Cells in colon organoids are γH2AX positive only when infected with the *pks*+ WT *E. coli*. Image representative of two independent replicates. Scale bars: 100 µm.
b. Comet assay of human colon organoid cells (each data point represents the mean of >80 cells) from three independent replicates shows more DNA damage in *pks*+ WT *E. coli*-infected cells. Data represent mean ± SD, $p < 0.05$, as calculated by one-way ANOVA and Tukey's test.

Source data are provided as a Source Data file.

Supplementary Figure 2:

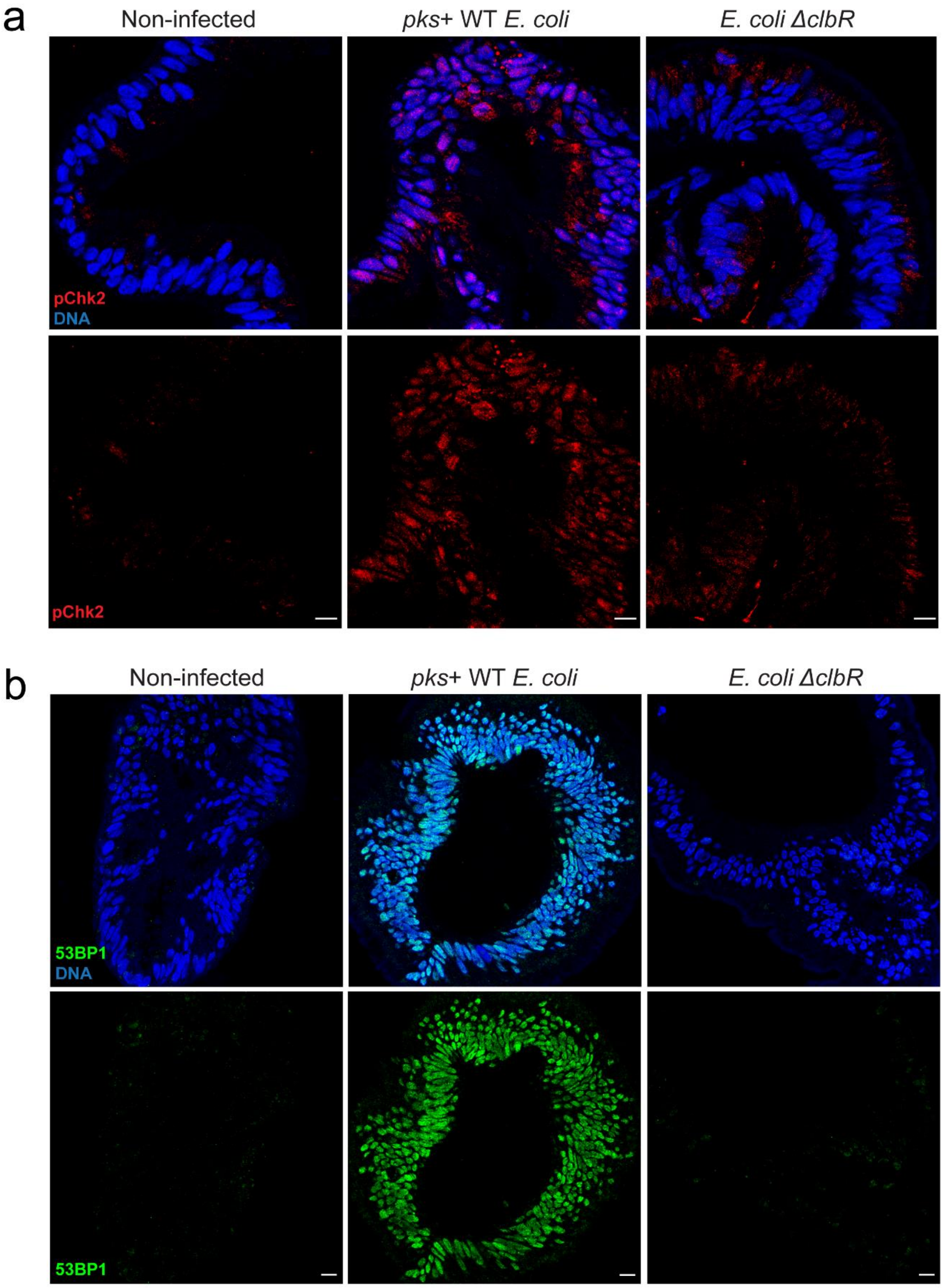

Supplementary Figure 2: *pks*+ *E. coli*-infected human colon organoids express DNA damage repair response proteins

a. Immunofluorescence staining for pChk2 (red). DAPI (blue) stains for DNA. Image representative of two independent replicates. Scale bars: 10 µm.
b. Immunofluorescence staining for 53BP1 (green). DAPI (blue) stains for DNA. Image representative of two independent replicates. Scale bars: 10 µm.

Supplementary Figure 3:

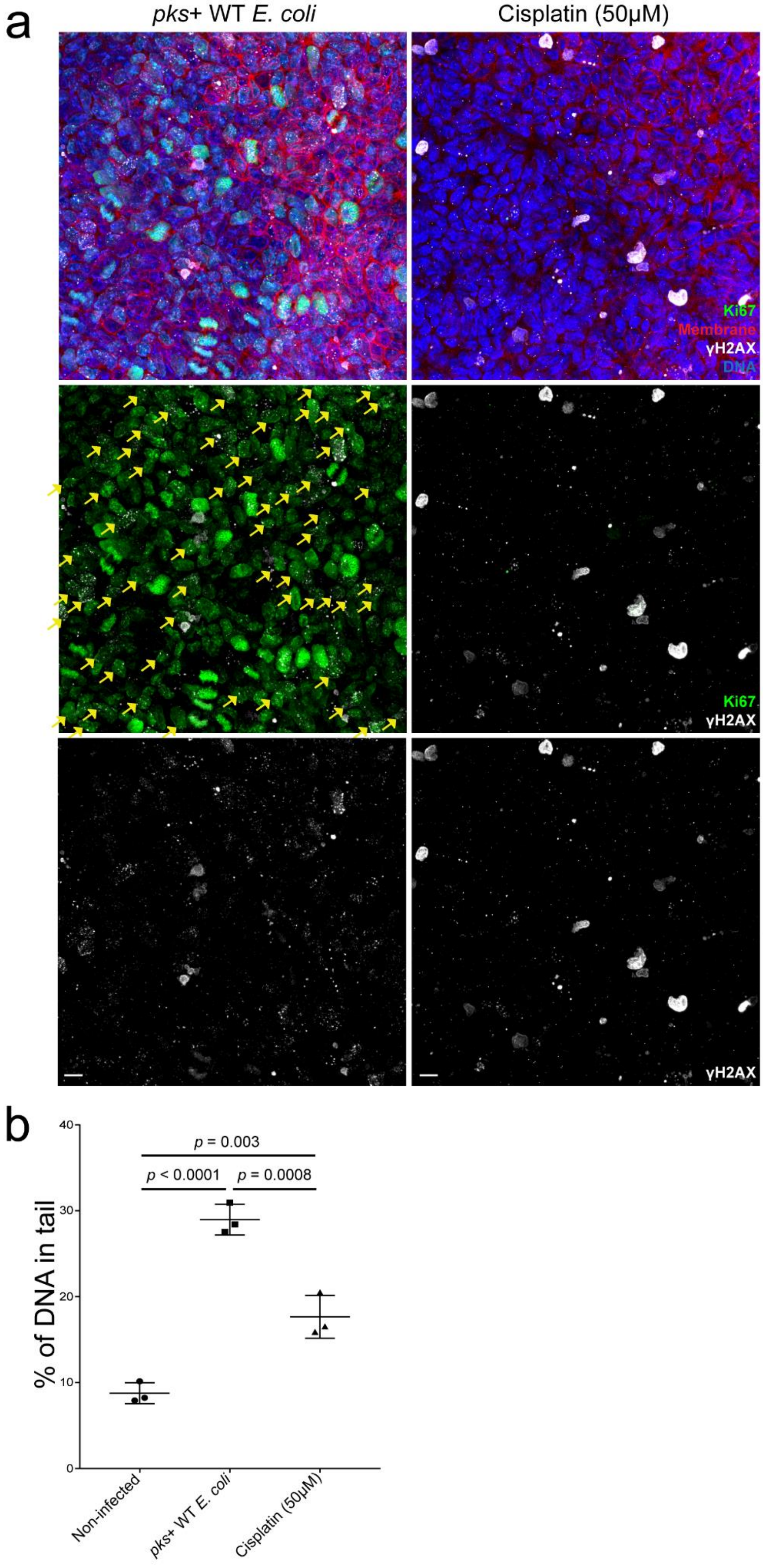

Supplementary Figure 3: *pks*+ *E. coli*-infected primary cells are double positive for γH2AX and Ki67

a. tdTomato (red) expressing colonic monolayer immunolabelled for γH2AX (white) and Ki67 (green). DAPI (blue) stains for DNA. Ki67 and γH2AX positive cells (yellow arrows) are observed for the monolayer infected with *pks*+ WT *E. coli* but not for cisplatin (50 µM) treated cells. Image representative of two independent replicates. Scale bars: 10 µm.
b. Comet assay of mouse colon organoid cells (each data point represents the mean of >80 cells) from three independent replicates shows more DNA damage in *pks*+ WT *E. coli*-infected cells compared to cisplatin (50 µM) treated cells. Data represent mean ± SD, $p < 0.05$, as calculated by one-way ANOVA and Tukey's test.

Source data are provided as a Source Data file.

Supplementary Figure 4:

a

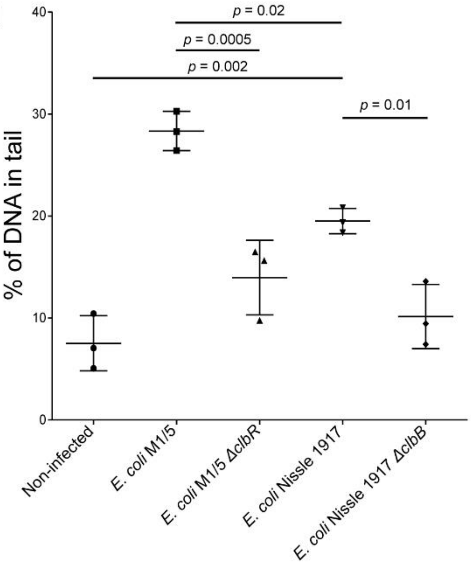

b

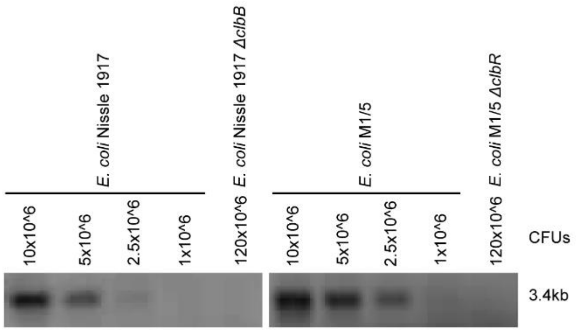

c

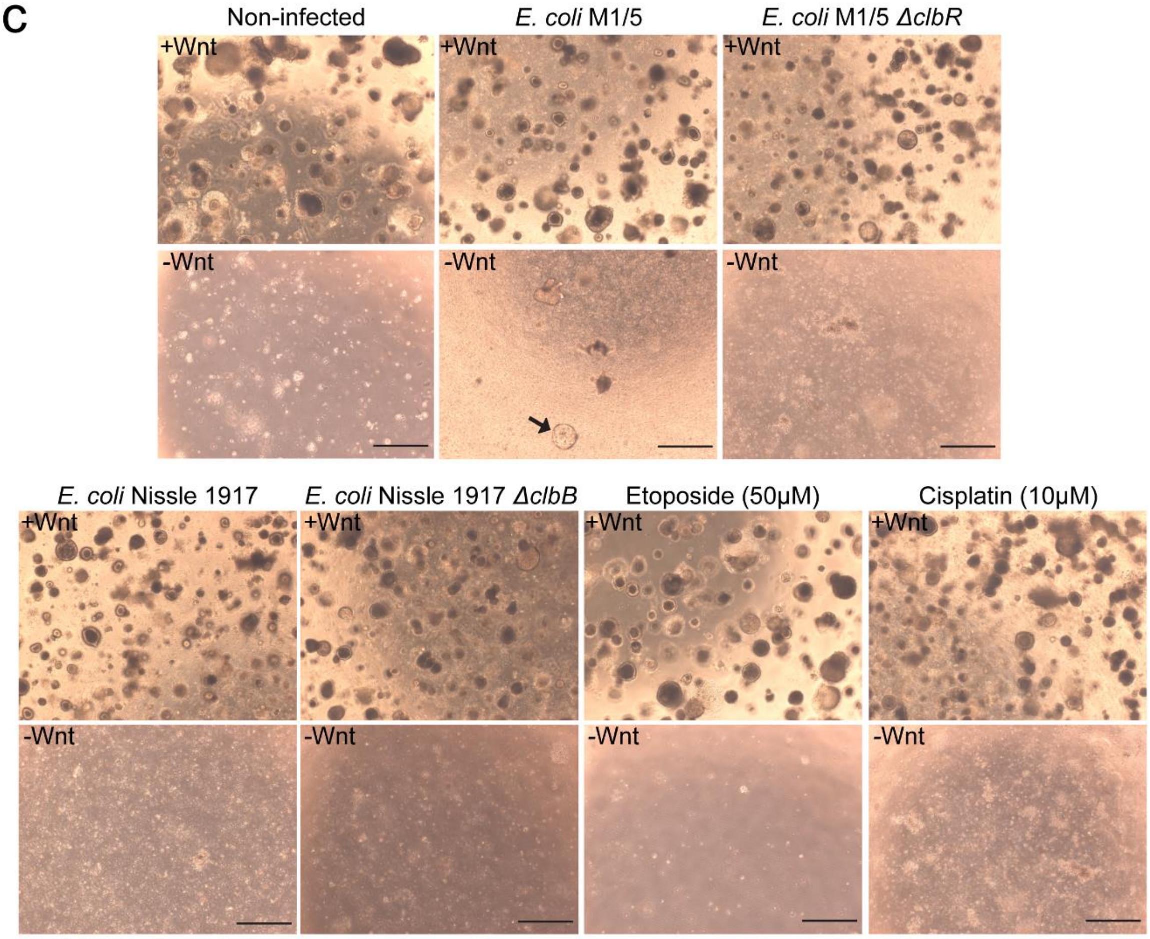

Supplementary Figure 4: *E. coli* Nissle 1917 causes less DNA damage and does not generate Wnt-independent organoids

a. Comet assay of mouse colon organoid cells (each data point represents the mean of >80 cells) from three independent replicates shows that *E. coli* Nissle 1917 causes DNA damage when compared to the non-infected condition. The DNA damage induced by *E. coli* M1/5 is significantly higher than that induced by *E. coli* Nissle 1917. Data represent mean ± SD, $p < 0.05$, as calculated by one-way ANOVA and Tukey's test.
b. DNA cross-linking assay shows the presence of cross-linked DNA at 3.4 kb for the indicated CFUs of *E. coli* Nissle 1917, *E. coli* Nissle 1917 *ΔclbB*, *E. coli* M1/5, and *E. coli* M1/5 *ΔclbR*. Image representative of three independent replicates.
c. Organoids grow without the addition of Wnt and CHIR99021 to the medium only for the *E. coli* M1/5-infected condition. Treatment with etoposide (50 µM) or cisplatin (10 µM) did not generate Wnt-independent organoids. For cisplatin, organoids did not grow in the presence of concentrations higher than 10 µM. Image representative of two independent replicates. Scale bars: 1 mm.

Source data are provided as a Source Data file.

Supplementary Figure 5:

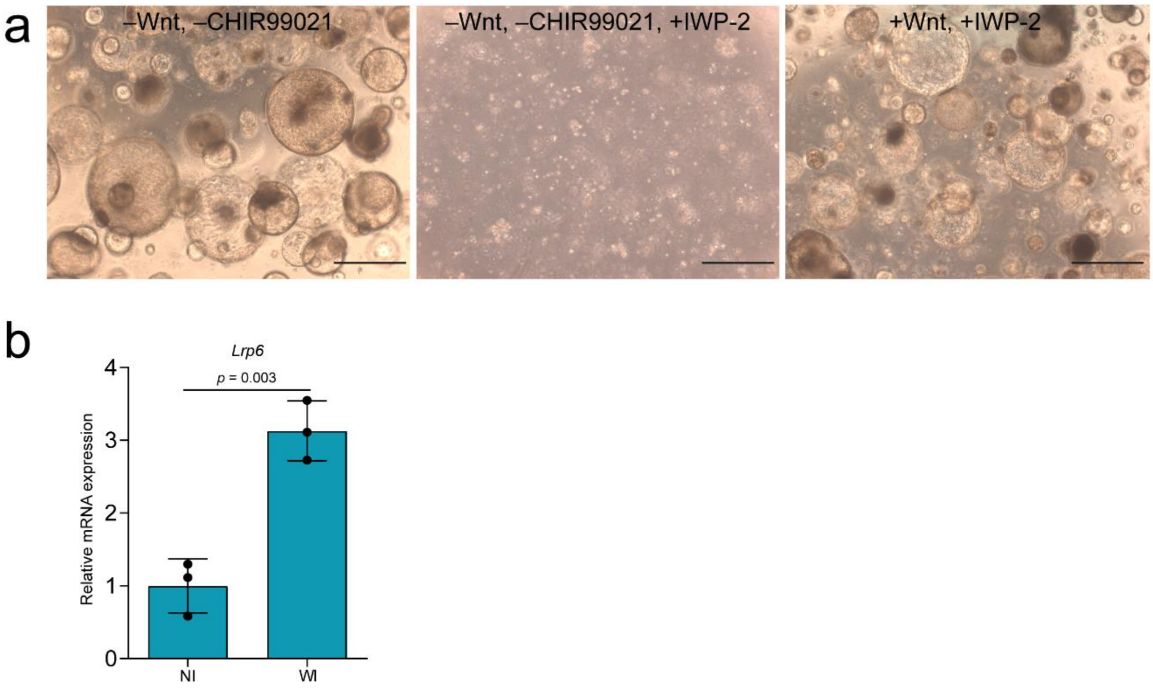

Supplementary Figure 5: Wnt-independent organoids do not grow in the presence of a porcupine inhibitor (IWP-2)

a. The Wnt-independent organoids do not grow in the presence of a porcupine inhibitor (IWP-2), and they can be rescued by providing exogenous Wnt. Image representative of three independent replicates. Scale bars: 1 mm.
b. qPCR data showing expression of *Lrp6* relative to *Gapdh* from three independent replicates. Data represent mean ± SD, $p < 0.05$, as calculated by two-sided unpaired Student's t-test. NI = Non-infected and WI = Wnt-independent.

Source data are provided as a Source Data file.

Supplementary Figure 6:

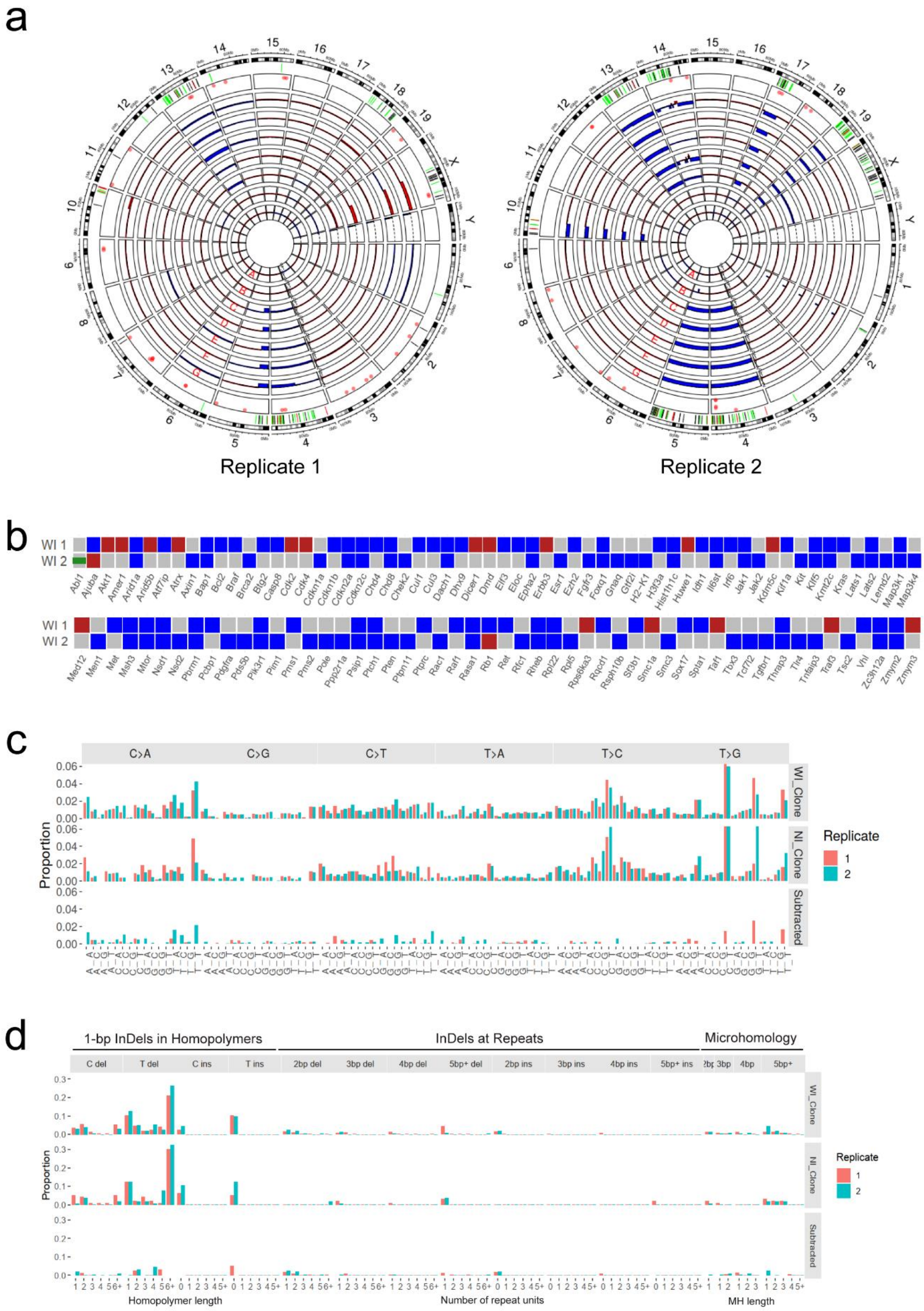

e

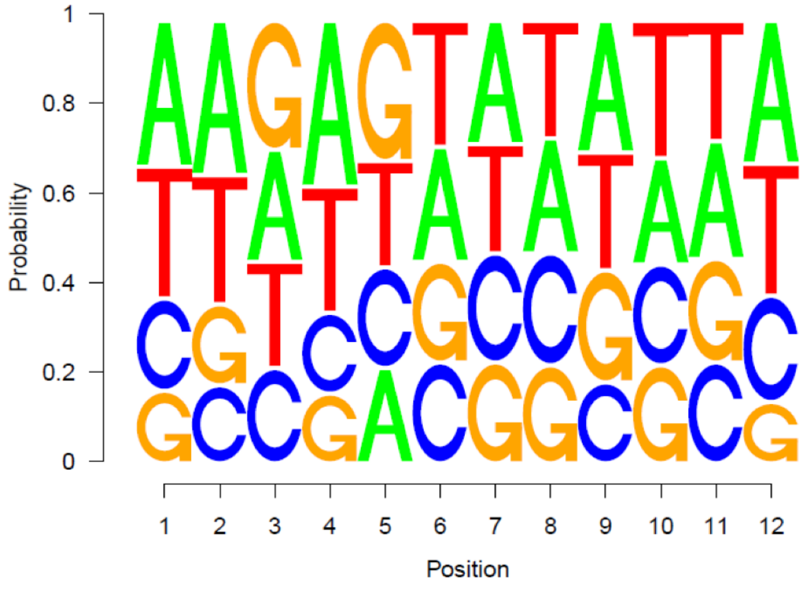

Supplementary Figure 6: Exome sequencing, gene mutations, mutational signature, and structural variant breakpoints

a. Circos plots showing CNV, SNV, indels, and mutated genes in the Wnt-independent organoids for Replicate 1 and 2 from whole-exome sequencing (WXS). Each section represents chromosomes 1 to 19, chromosome X, and chromosome Y. The innermost circles (A and B) represent non-infected organoid clones. C represents all organoids that grew without Wnt present in the medium after infection with *pks*+ WT *E. coli*. D to G represents Wnt-independent organoid clones derived from C. The blue (genomic loss), and red (genomic gain) regions represent CNV. The Y chromosome is lost in both replicates, but all other losses and gains of chromosomal regions are heterozygous. Each condition was compared to all the non-infected organoids of that respective replicate. A rainfall plot (red dots) shows the SNV and indel variations and the distances between them on the y-axis only for the Wnt-independent conditions. SNV/indel variant calls were done by pooling all Wnt-independent conditions. The green, orange, and black lines depict positions of mutated Wnt pathway genes, known cancer driver genes, and p53 pathway genes, respectively, in all Wnt-independent conditions.

b. Mutational status of genes excluding Wnt pathway genes that are frequently affected in colorectal cancer (pan-cancer drivers or tumour suppressors). Only genes with at least one mutational change (SNV, CNV) in the two replicates of Wnt-independent (WI) organoids are shown from whole-genome sequencing (WGS). Blue – genomic loss, red – genomic gain, and green bar – amino-acid sequence altering SNV/indel.

c. The proportion of single base substitution (SBS) 96-trinucleotide mutational changes (6 nucleotide changes in 16 trinucleotide contexts) among all SBSs for Wnt-independent (WI, top) and non-infected (NI, middle) clones. The bottom figure shows the excess of each trinucleotide change in Wnt-independent clones compared to the respective non-infected clones. Colours denote two independent replicates. Results obtained from WGS.

d. Mutational spectra of small indels in Wnt-independent (WI, top) compared to non-infected (NI, middle) clones from WGS. The bottom figure shows the excess of each indel class in Wnt-independent clones compared to the respective non-infected clones. Bars indicate the proportion of each indel type among all indels in this sample. Colours denote two independent replicates. Indel classes are, from left to right, indels of single C or T in C or T homopolymers of different lengths, longer (2-5+) indels in repetitive sequences, and those occurring at sites with overlapping microhomology of different lengths.

e. Sequence logo plot showing the relative contribution of nucleotides around 120 unique structural variant (SV) breakpoints (+/- 6 nucleotide positions) from two Wnt-independent (WI) samples analysed with WGS. Height of letters corresponds to relative contribution at each sequence position. SV breakpoint position is between positions 6 and 7. n=0 overlaps of SV breakpoints with AAWWTT or AAATT/AATTT.

Supplementary Figure 7:

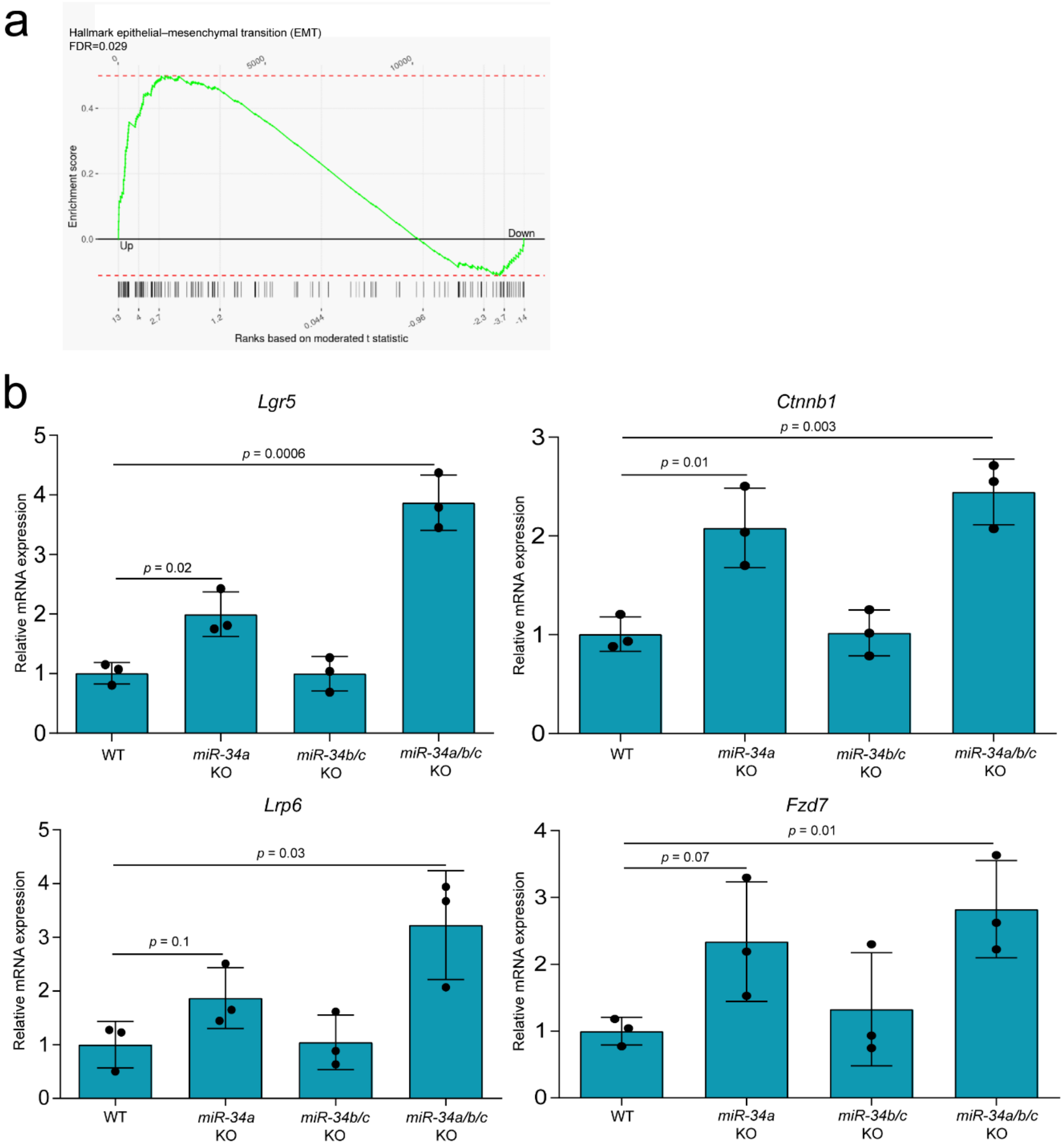

Supplementary Figure 7: *miR-34* KO organoids have upregulated Wnt signalling

a. Global comparison using gene set enrichment analysis between hallmark epithelial-mesenchymal transition (EMT) genes and RNA sequencing results from three replicates. Genes are ordered according to their differential expression in Wnt-independent vs. non-infected samples from left to right. Genes are marked by black bars below the plot. Plot line shows the running enrichment score.

b. qPCR data showing expression of *Lgr5*, β-catenin (*Ctnnb1*), *Lrp6*, and *Fzd7* relative to *Gapdh* for wild-type (WT) and *miR-34* KO mice from three independent replicates. Loss of *miR-34a* results in increased *Lgr5*, *Ctnnb1*, *Lrp6*, and *Fzd7* expression, but the loss of *miR-34a/b/c* causes an even higher expression of *Lgr5*, *Ctnnb1*, *Lrp6*, and *Fzd7*. Data represent mean ± SD, $p < 0.05$, as calculated by two-sided unpaired Student's t-test.

Source data are provided as a Source Data file.

Supplementary Table 1: Antibodies and dyes

IF – Immunofluorescence, WB – Western blot

Antibody	Supplier	Catalogue number	Dilution
**53BP1**	Novus Biologicals	NB100-304	IF – 1:500
**γH2AX**	Cell Signaling	2577	IF – 1:500
**β-catenin**	Cell Signaling	8480	IF – 1:300
**β-catenin**	BD Biosciences	610153	IF – 1:300
**Phospho-Chk2**	R&D Systems	AF1626	IF – 1:20
**E-cadherin**	BD Biosciences	610181	IF – 1:300
**MUC2**	Invitrogen	MA5-12345	IF – 1:50
**Ki67**	Cell Signaling	9129	IF – 1:250
**Ki67**	eBiosciences	11-5698-80	IF – 1:100
**p53**	Cell Signaling	2524	IF – 1:2000
**Phalloidin 546**	Invitrogen	A22283	IF – 1:200
**Hoechst**	Sigma	H6024	IF – 1:10000
**DAPI**	Roche	10236276001	IF – 1:500
**p53 (DO-1)**	Santa Cruz Biotechnology	SC-126	WB – 1:500
**β-actin**	Sigma-Aldrich	A5441	WB – 1:10000

Supplementary Table 2: Primers

Gene name	Primer sequence (5' – 3')
**Mouse Lgr5**	Forward – CCTACTCGAAGACTTACCCAGT<br>Reverse – GCATTGGGGTGAATGATAGCA
**Mouse Lef1**	Forward – ACAGGTCCCAGAATGACAGC<br>Reverse – TGGAGACAGTCTGGGGTTTC
**Mouse Fzd7**	Forward – GCTTCCTAGGTGAGCGTGAC<br>Reverse – AACCCGACAGGAAGATGATG
**Mouse Ctnnb1**	Forward – AGGGTGGGAATGGTTTTAGG<br>Reverse – GTGGCAAAAACATCAACGTG
**Mouse Car4**	Forward – CTGGGCAGCGTCTTTCC<br>Reverse – ATCTCCACTGTGTGTTGATTGTT

**Mouse Aqp8**	Forward – GTCCGAATACTGGGCTCCTG<br><br>Reverse – GTGTCCACCGCTGATGTTCC
**Mouse Lrp6**	Forward – TGATCGGAGAGGGTATGAGG<br><br>Reverse – AAGAACTCTTGGGCCTTGGT
**Mouse Gapdh**	Forward – TCACCATCTTCCAGGAGCG<br><br>Reverse – AAGCAGTTGGTGGTGCAGG
**Mouse pri-miR-34a**	Forward – CTGTGCCCTCTTGCAAAAGG<br><br>Reverse – GGACATTCAGGTGAGGGTCTTG
**Mouse pri-miR-34bc**	Forward – GGCAGGAAGGCTCCAGATG<br><br>Reverse – CCTCACTGTTCATATGCCCATTC
**Mouse Tubb**	Forward – AGTAAACCGTAGCCATGAGG<br><br>Reverse – CCTCCCAGAACTTAGCACC
