# Supplementary material for: Genomic aberrations after short-term exposure to colibactin-producing E. coli transform primary colon epithelial cells
Source: Nat Commun. 2021 Feb 12;12:1003. doi: 10.1038/s41467-021-21162-y (PMC7881031; doi:10.1038/s41467-021-21162-y)
Supplement: Supplementary file 2 — Reporting summary. [file 41467_2021_21162_MOESM2_ESM.pdf]

nature research

Corresponding author(s): Thomas F. Meyer

Last updated by author(s): Dec 15, 2020

Reporting Summary

Nature Research wishes to improve the reproducibility of the work that we publish. This form provides structure for consistency and transparency in reporting. For further information on Nature Research policies, seeAuthors & Referees and theEditorial Policy Checklist .

Statistics

For all statistical analyses, confirm that the following items are present in the figure legend, table legend, main text, or Methods section.

n/a	Confirmed	
☐	☒	The exact sample size (*n*) for each experimental group/condition, given as a discrete number and unit of measurement
☐	☒	A statement on whether measurements were taken from distinct samples or whether the same sample was measured repeatedly
☐	☒	The statistical test(s) used AND whether they are one- or two-sided<br>*Only common tests should be described solely by name; describe more complex techniques in the Methods section.*
☒	☐	A description of all covariates tested
☐	☒	A description of any assumptions or corrections, such as tests of normality and adjustment for multiple comparisons
☐	☒	A full description of the statistical parameters including central tendency (e.g. means) or other basic estimates (e.g. regression coefficient) AND variation (e.g. standard deviation) or associated estimates of uncertainty (e.g. confidence intervals)
☐	☒	For null hypothesis testing, the test statistic (e.g. *F*, *t*, *r*) with confidence intervals, effect sizes, degrees of freedom and *P* value noted<br>*Give P values as exact values whenever suitable.*
☒	☐	For Bayesian analysis, information on the choice of priors and Markov chain Monte Carlo settings
☒	☐	For hierarchical and complex designs, identification of the appropriate level for tests and full reporting of outcomes
☒	☐	Estimates of effect sizes (e.g. Cohen's *d*, Pearson's *r*), indicating how they were calculated

Our web collection on statistics for biologists contains articles on many of the points above.

Software and code

Policy information about availability of computer code

Data collection	Graphpad Prism 8, ImageJ 1.52, Comet Score software (TriTek 2.0)
Data analysis	Graphpad Prism was used for statistical analysis.<br>ImageJ was used to process immunofluorescence LIF files.<br>Comet Score was used to quantify the percentage of DNA in tail.<br>R-3.4 and corresponding packages were obtained from https://cran.r-project.org/. Additional R-packages were obtained from https://bioconductor.org<br>BWA v0.7.9 was obtained from http://bio-bwa.sourceforge.net/<br>Salmon v0.8.2 was obtained from https://combine-lab.github.io/salmon/<br>Strelka v1.0.11 was obtained from https://sites.google.com/site/strelkasomaticvariantcaller/<br>SnpEff v4.3k was obtained from http://snpeff.sourceforge.net/<br>GATK v3.4 and GATKv3.7 were obtained from https://software.broadinstitute.org/gatk/<br>CONTRA v2.0.8 was downloaded from http://contra-cnv.sourceforge.net/<br>ControlFREEC v10.8 was downloaded from https://github.com/BoevaLab/FREEC/releases/v10.8<br>SvABA (version 2018-05-02) was downloaded from https://github.com/walaj/svaba<br><br>All custom code used to analyze sequencing data is available from https://github.com/MPIIB-Department-TFMeyer/Iftekhar_Colibactin

For manuscripts utilizing custom algorithms or software that are central to the research but not yet described in published literature, software must be made available to editors/reviewers. We strongly encourage code deposition in a community repository (e.g. GitHub). See the Nature Research guidelines for submitting code & software for further information.

Data

Policy information about availability of data

All manuscripts must include a data availability statement. This statement should provide the following information, where applicable:

- Accession codes, unique identifiers, or web links for publicly available datasets
- A list of figures that have associated raw data
- A description of any restrictions on data availability

All RNA sequencing data is available from Gene Expression Omnibus (GEO, https://www.ncbi.nlm.nih.gov/geo/) with ID GSE140929. Whole-genome and exome sequencing data has been submitted to European Nucleotide Archive (ENA , https://www.ebi.ac.uk/ena) under accession PRJEB35529.
MSigDB v6.2 is available from http://www.gsea-msigdb.org/gsea/downloads_archive.jsp
Homologene build 68 is available from https://ftp.ncbi.nih.gov/pub/HomoloGene/
Gencode M12 mouse gene models were obtained from ftp://ftp.ebi.ac.uk/pub/databases/gencode/Gencode_mouse/release_M12/

Field-specific reporting

Please select the one below that is the best fit for your research. If you are not sure, read the appropriate sections before making your selection.

☒ Life sciences ☐ Behavioural & social sciences ☐ Ecological, evolutionary & environmental sciences

For a reference copy of the document with all sections, see nature.com/documents/nr-reporting-summary-flat.pdf

Life sciences study design

All studies must disclose on these points even when the disclosure is negative.

Sample size	No statistical methods were used to predetermine sample size. All experiments were performed with n = 3 biological replicates except where stated otherwise. Sample sizes were chosen based on available resources and community standards.
Data exclusions	No data were excluded from the results.
Replication	All attempts at replication were successful. All graphs represent data with at least three biological replicates. All images represent findings reproduced at least twice in the laboratory.
Randomization	Organoids were made from animals selected randomly. Cultured cells from each animal were randomly distributed to experimental conditions. No specific procedures were carried out for randomization for all experiments.
Blinding	The investigator was blinded for data collection and analysis of CFU counts, image analysis and for quantitative analysis.

Reporting for specific materials, systems and methods

We require information from authors about some types of materials, experimental systems and methods used in many studies. Here, indicate whether each material, system or method listed is relevant to your study. If you are not sure if a list item applies to your research, read the appropriate section before selecting a response.

Materials & experimental systems

n/a	Involved in the study	
☐	☒	Antibodies
☐	☒	Eukaryotic cell lines
☒	☐	Palaeontology
☐	☒	Animals and other organisms
☐	☒	Human research participants
☒	☐	Clinical data

Methods

n/a	Involved in the study	
☒	☐	ChIP-seq
☒	☐	Flow cytometry
☒	☐	MRI-based neuroimaging

Antibodies

Antibodies used	Rabbit anti-γH2AX (Cell Signaling, 2577), Rabbit anti-β-catenin (Cell Signaling, 8480), Rabbit anti-β-catenin (BD Biosciences, 610153), Rabbit anti-53BP1 (Novus Biologicals, NB100-304), Rabbit phospho-Chk2 (R&D Systems, AF1626), Mouse anti-E-cadherin (BD Biosciences, 610181), Mouse anti-Muc2 (Invitrogen, MA5-12345), Rabbit anti-Ki67 (Cell Signaling, 9129), Rat anti-Ki67 (eBiosciences, 11-5698-80), Mouse anti-p53 (Cell Signaling, 2524), Phalloidin 546 (Invitrogen, A22283), Hoechst (Sigma, H6024), DAPI (Roche, 10236276001), Mouse anti-p53 DO-1 (Santa Cruz Biotechnology, SC-126), Mouse anti-β-actin (Sigma-Aldrich, A5441), Sheep anti-mouse-HRP (Amersham, NA931)

Validation	Commercial antibodies were validated by the manufacturer for the respective applications; non-specific labelling was controlled for by labelling with secondary antibodies only; positive and negative controls were used for validation of each antibody where possible.

Eukaryotic cell lines

Policy information about cell lines

Cell line source(s)	Caco-2 (human colorectal adenocarcinoma) was obtained from ATCC (ATCC HTB-37).
Authentication	The cell line was not authenticated.
Mycoplasma contamination	Cell line tested negative for mycoplasma contamination.
Commonly misidentified lines (See ICLAC register)	No commonly misidentified cell lines were used in the study.

Animals and other organisms

Policy information about studies involving animals; ARRIVE guidelines recommended for reporting animal research

Laboratory animals	Experimental animals were male mice, 4-12 weeks of age of the following strains:<br>mTmG (JAX stock #007676, C57BL/6J background)<br>Trp53flox/flox (Jackson stock #008462, mixed C57BL/6J and C57BL/6N genetic background)<br>miR-34a KO, miR-34b/c KO, and miR-34a/b/c KO (Provided by Prof. Heiko Hermeking, C57BL6/SV129 background)<br>All mouse strains were described previously and original publications were cited for each strain, enabling readers to obtain more detailed information.
Wild animals	No wild animals were used in the study.
Field-collected samples	No field collected samples were used in the study.
Ethics oversight	All procedures involving animals were approved by the institutional and legal authorities at the Max Planck Institute for Infection Biology (Landesamt für Gesundheit und Soziales, Berlin).<br>The human colon biopsies and tissue samples were received from surgeries performed in Charité University Medicine, with prior approval of the ethics committee of the Charité University Medicine, Berlin (EA1/300/15).

Note that full information on the approval of the study protocol must also be provided in the manuscript.

Human research participants

Policy information about studies involving human research participants

Population characteristics	Tissue was donated by male patients aged 30 to 75
Recruitment	2016-2020
Ethics oversight	Ethics committee of the Charité University Medicine, Berlin

Note that full information on the approval of the study protocol must also be provided in the manuscript.
